# Transcriptional reprogramming underpins enhanced plant growth promotion by the biocontrol fungus *Trichoderma hamatum* GD12 during antagonistic interactions with *Sclerotinia sclerotiorum* in soil

**DOI:** 10.1111/mpp.12429

**Published:** 2016-07-24

**Authors:** Sophie Shaw, Kate Le Cocq, Konrad Paszkiewicz, Karen Moore, Rebecca Winsbury, Marta de Torres Zabala, David J. Studholme, Deborah Salmon, Christopher R. Thornton, Murray R. Grant

**Affiliations:** ^1^ Biosciences, College of Life and Environmental Sciences University of Exeter Geoffrey Pope Building, Stocker Road Exeter EX4 4QD UK; ^2^ Centre for Genome Enabled Biology and Medicine University of Aberdeen 23 St. Machar Drive Old Aberdeen AB24 3RY UK; ^3^ Sustainable Soils and Grassland Systems Department Rothamsted Research North Wyke Okehampton EX20 2SB UK; ^4^ Department of Biological Chemistry John Innes Centre Norwich Research Park Norwich NR4 7UH UK; ^5^ School of Life Sciences, Gibbet Hill Campus University of Warwick Coventry CV4 7AL UK

**Keywords:** biocontrol, plant growth promotion, RNA‐seq, *Sclerotinia sclerotiorum*, *Trichoderma hamatum*

## Abstract

The free‐living soil fungus *Trichoderma hamatum* strain GD12 is notable amongst *Trichoderma* strains in both controlling plant diseases and stimulating plant growth, a property enhanced during its antagonistic interactions with pathogens in soil. These attributes, alongside its markedly expanded genome and proteome compared with other biocontrol and plant growth‐promoting *Trichoderma* strains, imply a rich potential for sustainable alternatives to synthetic pesticides and fertilizers for the control of plant disease and for increasing yields. The purpose of this study was to investigate the transcriptional responses of GD12 underpinning its biocontrol and plant growth promotion capabilities during antagonistic interactions with the pathogen *Sclerotinia sclerotiorum* in soil. Using an extensive mRNA‐seq study capturing different time points during the pathogen–antagonist interaction in soil, we show that dynamic and biphasic signatures in the GD12 transcriptome underpin its biocontrol and plant (lettuce) growth‐promoting activities. Functional predictions of differentially expressed genes demonstrate the enrichment of transcripts encoding proteins involved in transportation and oxidation–reduction reactions during both processes and an over‐representation of siderophores. We identify a biphasic response during biocontrol characterized by a significant induction of transcripts encoding small‐secreted cysteine‐rich proteins, secondary metabolite‐producing gene clusters and genes unique to GD12. These data support the hypothesis that *Sclerotinia* biocontrol is mediated by the synthesis and secretion of antifungal compounds and that GD12's unique reservoir of uncharacterized genes is actively recruited during the effective biological control of a plurivorous plant pathogen.

## Introduction

There is an ever‐increasing interest in novel solutions to enhance crop yields. Agricultural intensification continues to be constrained by emerging, re‐emerging and endemic pathogens that currently account for ∼30% of losses in global crop production (Oerke and Dehne, 2004; Savary *et al*., 2012). Spiralling fertilizer costs and the associated financial carbon footprint of fossil fuel‐derived fertilizers mean that innovative solutions are needed to overcome suboptimal soil fertility if marginal land is to be effectively farmed (Hillier *et al*., 2009). Similarly, growing public anxiety of the environmental and health impacts of synthetic chemical additives is restricting the numbers of effective pesticides (Carvalho, 2006). These factors are coupled with increasing resistance to fungicides and public distrust in genetic modification (Frewer *et al*., 2004; Huesing *et al*., 2016; Magnusson and Hursti, 2002). Therefore, it is essential to identify innovative approaches to enhance productivity. Harnessing the natural growth promotion and biocontrol properties of plant‐beneficial rhizosphere microbes is an area with enormous potential to provide an increase in agricultural productivity, whilst minimizing inputs, waste and other negative impacts of agricultural intensification. The identification and exploitation of such biologics require approaches that combine multi‐scale studies of whole‐organism biology, genomics and chemistry.

The biological control and plant growth promotion (PGP) properties of *Trichoderma* species have been well documented (Hermosa *et al*., 2012; Matarese *et al*., 2012; Omann *et al*., 2012; Ryder *et al*., 2012). Diseases are controlled through direct hyperparasitism of rhizosphere pathogens (Benítez *et al*., 2004), through competition (Ryder *et al*., 2012), through the production of antimicrobial compounds (Steindorff *et al*., 2014; Vos *et al*., 2014), through the secretion of compounds that induce systemic resistance in plants (Lamdan *et al*., 2015) and, in many cases, most likely through a combination thereof (Howell, 2003). In addition, certain *Trichoderma* strains are able to improve the productivity of monocot and dicot plants by accelerating seed germination and root and shoot development (Chang *et al*., 1986; Yedidia *et al*., 2001), and by increasing resilience to abiotic stresses, such as drought (Bae *et al*., 2009; Donoso *et al*., 2008) and salinity (Brotman *et al*., 2013; Mastouri *et al*., 2010).

There is considerable agronomic interest in understanding the molecular mechanisms underpinning these beneficial processes. The transcriptional responses of three *Trichoderma* species with differing abilities to hyperparasitize *Rhizoctonia solani* are species specific, with distinct gene families and discrete biochemical processes being deployed by each fungus, despite the presence of orthologues across all three biocontrol species (Atanasova *et al*., 2013). Thus, the biocontrol capabilities of different *Trichoderma* species appear to be heavily dependent on specific transcriptional reprogramming events during antagonistic interactions. For example, during PGP, *T. virens* cultured hydroponically with maize or tomato roots deployed both common and plant‐specific transcriptional programmes, with important roles implicated for glycosyl hydrolases and transporters (Moran‐Diez *et al*., 2015). Plant–pathogen–hyperparasite interactions induce specific gene expression (Steindorff *et al*., 2014), including the over‐representation of transporters and carbohydrate‐active enzymes, similar to those identified during *T. virens* PGP (Moran‐Diez *et al*., 2015). Such robustness and flexibility in the deployment of different genome components to achieve biocontrol suggests that different modes of pathogen perception feed into a convergent network. Collectively, these findings imply that both biocontrol and PGP can utilize common biological pathways to synthesize and deliver bioactive antimicrobial and PGP compounds. Indeed, initial transcriptional responses of *T. harzianum* grown in the presence of tomato plants, chitin (an integral component of the fungal cell wall) or a simple glucose medium showed much greater similarity between plant and chitin, rather than glucose, treatments, inferring that, during initial establishment, common molecular mechanisms apply to the processes of PGP and biocontrol (Samolski *et al*., 2009).

The majority of *Trichoderma* species investigated to date usually exhibit either PGP or biocontrol properties. Notably, *T. hamatum* strain GD12 (hereafter referred to as GD12) is a rare example that exhibits both properties (Ryder *et al*., 2012; Studholme *et al*., 2013). In lettuce microcosms containing low‐pH, nutrient‐poor peat, GD12 elicits both the biocontrol of root‐infecting pathogens and PGP (Ryder *et al*., 2012; Studholme *et al*., 2013). Genome sequencing of GD12 revealed a strikingly expanded genome with over 4500 predicted proteins without orthologues in four closely related *Trichoderma* species, including a large percentage of the predicted secretome—classically comprising small secreted cysteine‐rich proteins (SSCRPs). Some SSCRPs are likely to function as potential effector molecules that collaborate in biocontrol and PGP. Consistent with this, in other *Trichoderma* species, SSCRPs have been shown to be up‐regulated during PGP (Moran‐Diez *et al*., 2015), during induction of systemic resistance (Lamdan *et al*., 2015) and during biocontrol (Atanasova *et al*., 2013). Thus, the expanded repertoire of SSCRPs unique to GD12 may contribute to its dual biocontrol and PGP properties.

In this study, we compared the transcriptional response of GD12 in peat during lettuce PGP with that induced during its biocontrol of the pathogen *Sclerotinia sclerotiorum* over 15 days, sampling at six time points. Using mRNA‐seq, we captured the extent of transcriptional reprogramming in both GD12 and GD12–*S. sclerotiorum* mixed microcosms during the PGP and biocontrol processes, and showed that antagonistic interaction between *S. sclerotiorum* and GD12 activates unique and extensive transcriptional reprogramming early in the interaction, not seen under PGP conditions. Of particular note, underpinning these responses are specific subsets of both SSCRPs and predicted secondary metabolite‐associated gene clusters, indicative of important roles for both during PGP and disease amelioration.

## Results and Discussion

### PGP of lettuce by *T. hamatum* strain GD12 is increased during antagonism of *S. sclerotiorum*

In this study, we investigated the transcriptional reprogramming in *T. hamatum* strain GD12 during its antagonism of the soil pathogen *S. sclerotiorum*. GD12 biocontrol of *S. sclerotiorum* not only abrogates post‐emergence pathogenesis of lettuce, but, remarkably, further enhances lettuce growth promotion in comparison with GD12 alone (Studholme *et al*., 2013).

**Figure 1:**
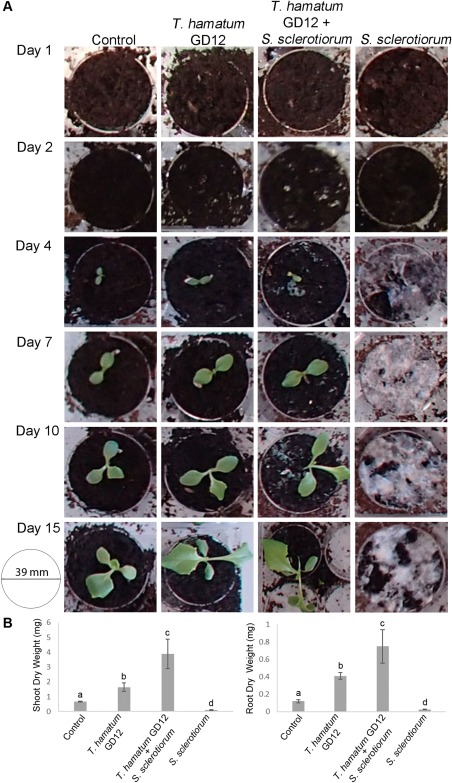
Summary overview of individual lettuce plants showing plant growth promotion and biocontrol of *Sclerotinia sclerotiorum* by *Trichoderma hamatum* GD12 through the 15‐day experimental period. (A) Photographs of wells containing a single representative lettuce plant growing in peat treated with *T. hamatum* GD12 only, *S. sclerotiorum* only, *T. hamatum* GD12 and *S. sclerotiorum* or with no treatment (Control). The diameter of each well is 39 mm. A full overview is presented in Fig. S1. (B) Quantification of plant growth promotion by *T. hamatum* GD12 in the presence of *S. sclerotiorum*. Dry weights of lettuce shoots and roots were measured after 14 days of growth in peat supplemented with the treatments presented in (A). Each bar is the mean dry weight per plant of three experimental replicates, each containing (25) replicates (plants) ± standard errors. Bars with different letters are significantly different from one another at *P* < 0.05.

A peat microcosm time course was set up in six‐well plates containing peat only (control), peat with GD12 only, peat with *S. sclerotiorum* only or peat with GD12 and *S. sclerotiorum*, each well containing a single lettuce seedling. Plates were randomized daily. At 1, 2, 4, 7, 10 and 15 days, random triplicate samples for each treatment (each sample comprising two pooled wells) were harvested and immediately snap frozen in liquid nitrogen (for the experimental design, see Fig. S1 in Supporting Information). Inoculation of peat with *T. hamatum* GD12 showed significant PGP of lettuce across the 15‐day time course compared with lettuce grown in peat alone (Fig. 1A). In addition, antagonistic interactions following amendment with *S. sclerotiorum* led to further enhancement of PGP (Fig. 1A), as reflected in the significant increases in dry weight in both roots and shoots during the antagonistic interaction compared with GD12 alone (Fig. 1B). As expected, no lettuce germinated in the peat microcosms containing *S. sclerotiorum* only.

### Identification of differentially expressed genes (DEGs) differs significantly between DESeq2, CuffDiff2 and edgeR

**Figure 2:**
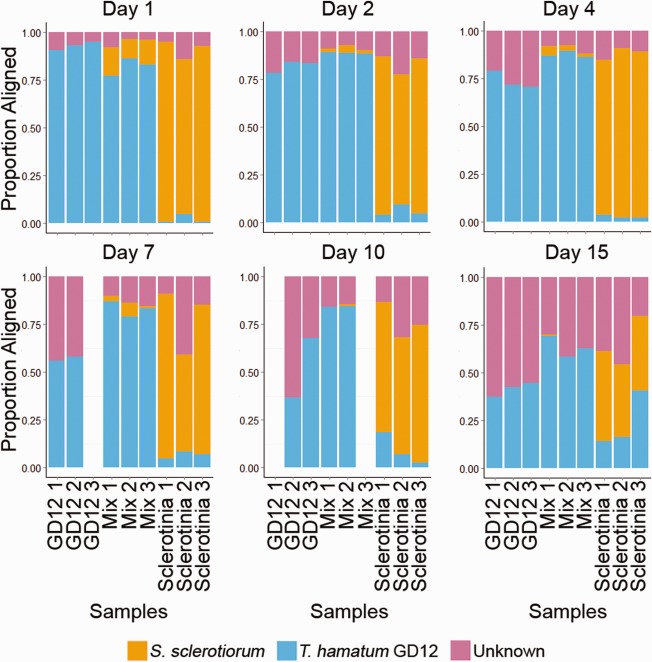
Summary of mRNA‐seq read distributions across the time course. Proportion of RNA reads at 1, 2, 4, 7, 10 and 15 days aligned to each reference genome. Blue, proportion of reads aligned to the *Trichoderma hamatum* GD12 reference. Orange, proportion of unmapped reads then aligned to the *Sclerotinia sclerotiorum* 1980 reference. Pink, proportion of unmapped reads.

To assess the transcriptional response of GD12 and *S. sclerotiorum* during plant–pathogen–hyperparasite interactions, RNA was extracted directly from the frozen microcosm samples. Bar‐coded cDNA libraries were sequenced using Illumina 100‐bp paired‐end reads. The resulting processed RNA reads were aligned to the GD12 reference genome (Studholme *et al*., 2013), and unmapped reads were then aligned to the *S. sclerotiorum* reference isolate 1980 (Amselem *et al*., 2011). As expected, the majority of RNA reads aligned to the *T. hamatum* reference (Studholme *et al*., 2013) in both the GD12‐only and mixed‐species microcosms (MSMs) (Fig. 2). We hypothesize that the increased proportion of GD12 reads seen at 4–7 days in the MSMs is accounted for by the stimulation of antimicrobial activity in the presence of *S. sclerotiorum*. Similarly, *S. sclerotiorum* formed the majority of reads in the *S. sclerotiorum*‐only microcosms, and represented a declining proportion of the RNA population in the MSMs as the time course progressed, consistent with activation of the GD12 biocontrol. At 1 day, *S. sclerotiorum* reads accounted for approximately 10%–20% of total reads in the MSMs, and this had declined to less than 5% by 2 days. Notably, from 2 days, there was a much larger proportion of reads aligning to GD12 in the MSMs than in the GD12‐only microcosms, indicating enhanced transcriptional activity as a result of antagonism (Fig. 2). It should be noted that the increasing proportion of GD12 reads in the *S. sclerotiorum*‐only samples, notable at 15 days, was exacerbated by the alignment strategy, which first aligned reads to the GD12 genome, and then to the *S. sclerotiorum* genome, and thus any *Sclerotinia* reads belonging to genes that are common with or highly similar to GD12 will first be identified as *Trichoderma* reads. Across all samples, on average, 4% of reads aligned to both the GD12 and *Sclerotinia* genomes. Table S1 (see Supporting Information) contains full read statistics and read alignments to each specific genome.

All samples showed a proportion of RNA that did not align to either genome. This proportion increased over the duration of the experiment and most likely represents natural colonization by airborne organisms in the controlled environment chamber over time. Notable was the increase in the proportion of unknown reads over time and, in particular, the larger proportion of these evident as early as 4 days in MSMs, in which cryptic chemistries activated by GD12–*S. sclerotiorum* antagonistic interactions are present.

Of the 54 RNA extractions, the level of recovery from two replicates, 7‐day GD12 replicate 3 and 10‐day GD12 replicate 1, was insufficient to prepare high‐quality mRNA‐seq libraries. On sequencing the 52 replicates, a single mixed species replicate at 10 days was excluded from further analysis because of the unusually high proportion of reads mapping to the *S. sclerotiorum* genome. This sample had been previously flagged, as it corresponded to a sample well that contained a single plant with atypically less growth (circled in Fig. S1), indicative of an isolated example of failed biocontrol. The absence of these data points is accounted for by the analysis software and does not therefore have a negative effect on the dataset.

Our primary focus for this study was how *S. sclerotiorum* modified the transcriptional response of GD12 during antagonistic interactions in peat. The strategy was to compare the expression profiles during this antagonistic response with those of GD12 alone which underpins PGP. Genes with significantly reduced expression or no expression in GD12‐only microcosms would be assigned as up‐regulated in MSMs, and vice versa for those genes identified as up‐regulated in the GD12‐only microcosms. Analysis of these differences is predicted to provide an insight into how GD12 deploys its significantly expanded genome in biocontrol.

We first analysed the differences in the transcriptional response of GD12 and *S. sclerotiorum* using three separate differential expression analysis software packages. HTSeq (Anders *et al*., 2014) was used for the quantification of reads at each predicted gene location, which were then employed for DESeq2 (Love *et al*., 2014) and edgeR (Robinson *et al*., 2010) analyses. In addition, Cufflinks (Trapnell *et al*., 2010) was used to quantify transcripts, which were then analysed by CuffDiff2 (Trapnell *et al*., 2013). All three methods used a significance threshold of <0.05 for either the adjusted *P* value or *q* value, representing a 5% false discovery rate, corrected for multiple testing using the Benjamini–Hochberg method (Benjamini and Hochberg, 1995).

**Figure 3:**
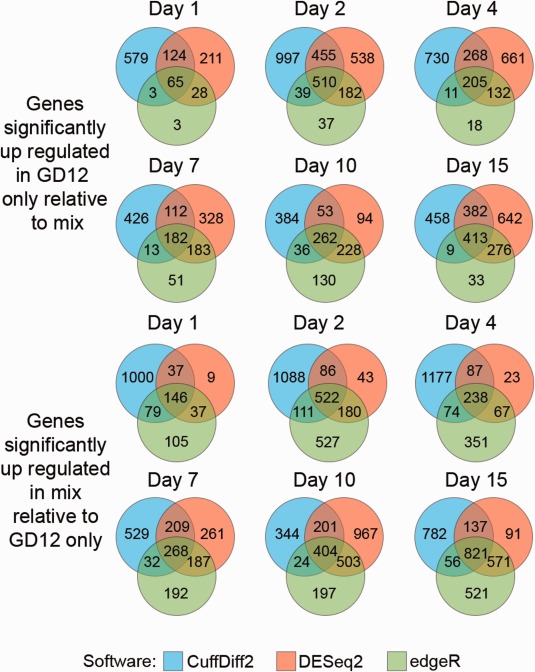
Classification of differentially expressed genes using three differential expression analysis software packages. Number of differentially expressed genes identified by CuffDiff2 (blue), DESeq2 (red) and edgeR (green) at each time point. The top panel shows the number of genes with significantly higher expression in the *Trichoderma hamatum* GD12‐only microcosms, whereas the bottom panel shows the number of genes with significantly higher expression in the mixed‐species microcosms.

The DEGs relative to each condition differed strikingly depending on the software chosen (Fig. 3). This appears to be a consequence of the different statistical models used between software packages. CuffDiff2 applies a negative binomial model, followed by computation of a *t*‐like statistic based on fragments per kilobase per million mapped read (FPKM) values (Trapnell *et al*., 2010). DESeq2 and edgeR both use raw count data. However, DESeq2 uses a negative binomial model with a general linear model for comparison (Love *et al*., 2014), whereas edgeR uses a similar over‐dispersed Poisson model to explain variation, followed by an empirical Bayes model (Robinson *et al*., 2010). It is surprising that such remarkable differences are generally not considered when undertaking mRNA‐seq studies, as these statistical software packages have been compared previously using data from the well‐annotated *Saccharomyces cerevisiae* genome (Nookaew *et al*., 2012). Similar to the results seen in Fig. 3, the study by Nookaew *et al*. (2012) showed large variations in the number of DEGs; however here edgeR identified a much higher number of genes, whereas Nookaew *et al.* (2012) show CuffDiff2 identified the largest number of DEGs. Despite these differences, the overall biological interpretations based on gene ontology (GO) did not differ between software programs (Nookaew *et al*., 2012).

Log‐fold changes, as calculated by DESeq2 and edgeR, can easily be compared because the gene model used by each program is identical. In our data, these log‐fold changes showed highly significant correlations (Pearson correlation > 0.97) despite differences in the number of DEGs identified (Fig. S2, see Supporting Information). Comparisons with CuffDiff2 are more complex as it only uses gene models as a guide and exact locations of transcripts are predicted *de novo*. In addition to our 12 591 gene predictions, Cufflinks predicted a further 8076 transcripts and frequently altered gene coordinates, thus contributing to the differences observed in Fig. 3.

For all subsequent analyses, we chose to use DESeq2 for the following reasons. First, CuffDiff2 predicts *de novo* transcript coordinates that do not match the FGenesH predictions in the GD12 reference (Studholme *et al*., 2013), notably the bespoke predictions of SSCRP‐encoding genes associated with the GD12 secretome. Second, despite finding similar biological ontologies between DEGs (Nookaew *et al*., 2012), it has been reported that edgeR produces a higher number of false positives (Zhang *et al*., 2014). To use the intersection of all three programs would multiply all biases; thus, based on the arguments above, the more conservative DESeq2 was used to focus on the most robustly identified differential signals.

### *Trichoderma hamatum* GD12 has a distinct transcriptional profile when confronted with *S. sclerotiorum*

**Figure 4:**
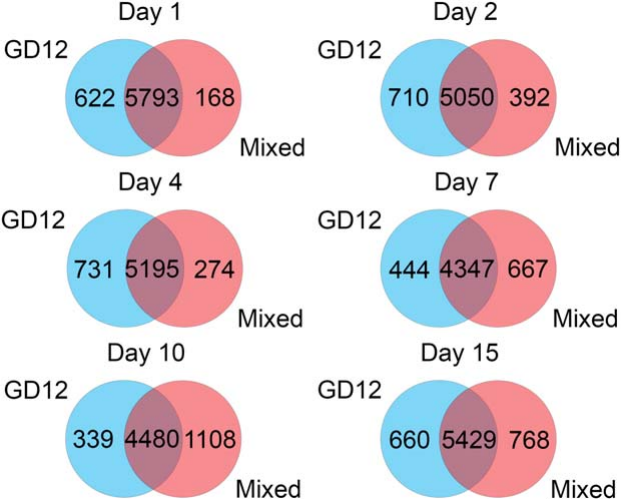
Treatment‐specific gene dynamics over the experimental time course. Number of genes expressed (normalized count ≥ 25) in either the *Trichoderma hamatum* GD12‐only microcosms (blue) or the mixed‐species microcosms (red) at each time point, as determined by DESeq2.

We defined ‘expressed’ as having a DESeq2‐normalized count of ≥25. Using this criterion, we identified ∼50% of the 12 591 predicted genes in GD12 and MSM treatments as being expressed during the time course. Figure 4 summarizes the differences in expression between GD12 and GD12/*S. sclerotiorum* peat amendments over the 15‐day time course. The core shared transcriptome between treatments comprised between ∼4300 and ∼5800 genes. A greater percentage of expressed genes were present in GD12 treatments between 1 and 4 days (Fig. 4). By contrast, between 7 and 15 days, the MSM treatment had a larger proportion of expressed genes, numerically representing nearly 25% of the core transcriptome at 10 days (Fig. 4). The *S. sclerotiorum* transcriptional profile was not the primary objective of this study. We initially undertook the analysis of differential gene expression in *S. sclerotiorum*; however, the dramatically reduced number of reads in the MSMs compared with the monoculture, because of aggressive biocontrol by GD12, meant that any differential expression analysis of the *S. sclerotiorum* transcriptome would not be statistically valid. The summary of the read numbers and raw data is available in Table S1 (GEO accession number GSE67909).

**Figure 5:**
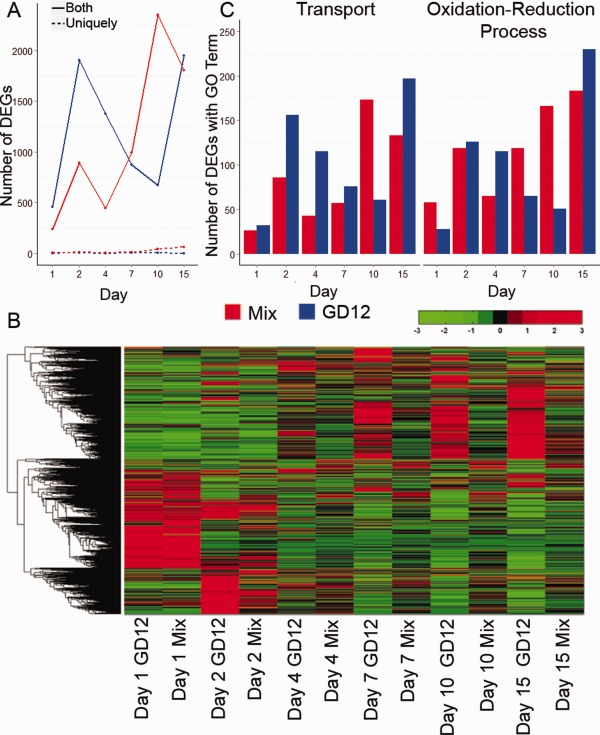
Comparison of the transcriptional dynamics of *Trichoderma hamatum* GD12 peat microcosms with mixed peat microcosms of *T. hamatum* GD12 and *Sclerotinia sclerotiorum*. (A) The transcriptional response in GD12‐only microcosm (blue) peak at 2 and 15 days, and the transcriptional response in the mixed‐species microcosm (red) peak at 2 and 10 days. The broken lines show that the number of differentially expressed genes (DEGs) with counts solely in one sample, with no expression in the other, was very few for both GD12‐only and mixed‐species microcosms. (B) The transcriptional response of *T. hamatum* GD12 is highly dynamic. Genes clustered by normalized counts using clustergram in MATLAB. Red shows a high level of expression, whereas green shows a low level of expression. (C) The percentage of DEGs associated with the gene ontology (GO) terms ‘transport’ and ‘oxidation–reduction’ is higher in mixed‐species microcosms at initial time points up to 4 days.

DEGs identified between GD12 only and MSMs, and their annotations (Blast2GO predictions; Conesa *et al*., 2005), are summarized in Table 1 and catalogued in Supporting Material S1–S4. The transcriptional reprogramming is primarily the result of differential expression rather than the induction of novel transcripts, as illustrated in Fig. 5A, which captures the strikingly sharp increase in expression between 1 and 2 days following GD12 amendment. Previous studies conducted in soil‐less systems over short time periods have shown the early transcriptional reprogramming of *T. harzianum* during the initial stages of biocontrol (Samolski *et al*., 2009; Steindorff *et al*., 2014). By contrast, our experiments conducted in more complex peat microcosms and over a more sustained experimental period revealed a biphasic dynamic with a further significant number of DEGs evident in MSMs at 10 and 15 days (Fig. 5A) when differential PGP between treatments was evident (Fig. 1). This later transcriptional response may involve the induction of components associated with PGP or, possibly, a biocontrol response to the establishment of natural communities, as inferred from the unmapped reads in Fig. 2. As illustrated above, the majority of DEGs were present under both conditions. Only seven genes were uniquely expressed in GD12 only, whereas more than 15 times as many unique genes (114) were induced in MSMs over the 15‐day time course (Table 1 and Fig. 5A). Predicted gene annotations are provided in Supporting Material S3 and S4; the determination of the function of these genes may facilitate a better understanding of biocontrol mechanisms.

**Table 1:** Number of differentially expressed GD12 genes identified by DESeq2 at each time point in GD12‐only or GD12 + *Sclerotinia sclerotiorum* treatments, and genes which are uniquely present to the treatment [normalized count = 0, false discovery rate (FDR) = 5%, Benjamini–Hochberg correction).

Time point	Number of genes up‐regulated in peat in GD12 only	Number of genes up‐regulated in peat in GD12‐only where lower expression is 0	Number of genes up‐regulated in peat in mix	Number of genes up‐regulated in peat in mix where lower expression is 0
Day 1	454	1	238	0
Day 2	1905	2	891	7
Day 4	1378	0	442	3
Day 7	873	2	993	10
Day 10	669	2	2351	42
Day 15	1952	0	1808	58

Hierarchical clustering showed that the transcriptional response within and across treatments over the 15 days was remarkably dynamic (Fig. 5B), with only nine genes in GD12‐only microcosms and 22 genes during antagonism challenge, of the ∼6000 expressed genes, significantly up‐regulated at all time points (Table 2). These nine GD12‐specific genes encode, amongst others, methyltransferases, a malic acid transporter and a potassium uptake protein (Table 3). The predicted functions of the 22 genes which were persistently up‐regulated in the MSM encompass both components of antagonism and those required for a protective strategy to restrict self‐damage to GD12 occurring from antimicrobial compounds released during antagonism. Four are predicted to encode transporters, including one associated with resistance to the antifungal triazole, fluconazole. Two genes are predicted to encode dienelactone hydrolases, involved in the degradation of catechol by bacteria (Schmidt and Knackmuss, 1980; Schlömann *et al*., 1993), and a pyoverdine dityrosine biosynthesis gene. In bacteria, pyoverdine acts as an iron siderophore, enabling iron sequestration from the environment (Meyer, 2000). Notably, iron‐binding siderophores have been shown to be important for the saprotrophic lifestyle of *Trichoderma* species (Anke *et al*., 1991; Hoyos‐Carvajal *et al*., 2009; Renshaw *et al*., 2002). In addition, of three *Streptomyces* isolates producing siderophores tested for their PGP capabilities, isolates producing siderophores were found to have the greatest increase in plant biomass (Verma *et al*., 2011).

**Table 2:** Number of differentially expressed GD12 genes at concurrent time points during GD12‐only plant growth promotion (PGP) (A) and mixed‐species antagonism (B) identified by DESeq2 at each time point.

(A) GD12 only	1 day	1 and 2 days	1, 2 and 4 days	1, 2, 4 and 7 days	1, 2, 4, 7 and 10 days	1, 2, 4, 7, 10 and 15 days
	454	51	21	11	9	9
		2 days	2 and 4 days	2, 4 and 7 days	2, 4, 7 and 10 days	2, 4, 7, 10 and 15 days
		1905	293	47	23	22
			4 days	4 and 7 days	4, 7 and 10 days	4, 7, 10 and 15 days
			1378	306	149	135
				7 days	7 and 10 days	7, 10 and 15 days
				873	399	370
					10 days	10 and 15 days
					669	562
						15 days
						1952
(B) Mixed species	1 day	1 and 2 days	1, 2 and 4 days	1, 2, 4 and 7 days	1, 2, 4, 7 and 10 days	1, 2, 4, 7, 10 and 15 days
	238	87	48	29	26	22
		2 days	2 and 4 days	2, 4 and 7 days	2, 4, 7 and 10 days	2, 4, 7, 10 and 15 days
		891	187	104	98	88
			4 days	4 and 7 days	4, 7 and 10 days	4, 7, 10 and 15 days
			442	220	210	176
				7 days	7 and 10 days	7, 10 and 15 days
				993	804	613
					10 days	10 and 15 days
					2351	1258
						15 days
						1808

Based on a 5% false discovery rate, using a Benjamini–Hochberg multiple comparison correction.

**Table 3:** Genes significantly up‐regulated in either GD12‐only or mixed‐species microcosms at every time point.

Up‐regulated during GD12 only			Up‐regulated during mixed species		
Gene name (ANCB0XXXX)	Log_2_ fold change at day one	blastx hit	Gene name (ANCBXXXX)	Log_2_ fold change at day one	blastx hit
1006842.1:599‐2594	5.19	NAD‐dependent epimerase dehydratase	1009877.1:936‐3483	6.19	
1003985.1:771‐3990	1.98	Malic acid transport protein	1007557.1:3442‐5242	5.65	
1002344.1:7484‐8547	1.85	UbiE/COQ5 methyltransferase	1005725.1:65‐1614	4.96	ABC multidrug
1010824.1:460‐1942	1.57		1011666.1:16‐764	4.62	
1002225.1:1802‐2938	1.47	GNAT family protein	1010015.1:968‐2443	4.48	Pyoverdine dityrosine biosynthesis
1006475.1:142‐1395	1.20	Methyltransferase‐like protein	1011665.1:29‐942	4.43	
1013852.1:19‐1423	1.19	Tam domain	1009227.1:295‐2134	3.7	Dienelactone hydrolase family protein
1002254.1:150‐2104	1.18		1013178.1:264‐4974	3.67	Transferase family protein
1003305.1:15‐928	0.92	Potassium uptake protein	1013143.1:5756‐7036	3.56	Methyltransferase domain‐containing protein
			1004667.1:16‐788	3.55	
			1001608.1:249‐1661	3.47	Mitochondrial phosphate carrier protein
			1014508.1:600‐2990	3.39	Short‐chain dehydrogenase reductase
			1013443.1:1767‐2642	3.33	Fluconazole resistance protein 1
			1002135.1:4185‐5616	3.24	
			1010016.1:125‐2333	2.92	Major facilitator superfamily transporter
			1009878.1:815‐3132	2.91	Family regulatory protein
			1000041.1:739‐1491	2.50	FAD‐linked oxidase
			1010706.1:2182‐4056	2.40	GNAT family
			1009777.1:2530‐3653	2.08	Dienelactone hydrolase family protein
			1005944.1:2672‐3858	1.86	FAD‐dependent oxidoreductase
			1005794.1:2226‐3132	1.83	Aryl‐alcohol dehydrogenase
			1003990.1:109‐1714	1.21	Major facilitator superfamily transporter

FAD, flavin adenine dinucleotide; NAD, nicotinamide adenine dinucleotide; GNAT, GCN5‐related N‐acetyltransferase.

The potential functional roles of DEGs were investigated using GO terms. Although both transport and oxidation–reduction (redox) process terms were strongly over‐represented across all time points under both conditions (Fig. 5C), redox processes were significantly more abundant in the MSMs at 1, 7 and 10 days. Strikingly, at 1 day, there were twice as many redox‐associated DEGs in the MSM compared with GD12 only. This suggests that redox components are actively recruited during early antagonistic events. There were also contrasting patterns in ‘transport’ process DEGs, with a marked over‐representation in the GD12‐only microcosms at 2 days, whereas this signature was not observed until 10 days in MSMs (Fig. 5C), suggestive of initial active prioritization of *S. sclerotiorum* biocontrol over PGP observed at 10 days (Fig. S1).

### Quantitative polymerase chain reaction (qPCR) validation of early DEGs

Given the gross differences seen in the different algorithms for predicting DEGs, we validated the DESeq2‐predicted expression profiles of selected genes at day 2, or days 2 and 15 post‐treatment, by quantitative reverse transcription‐polymerase chain reaction (qRT‐PCR) (for primers, see Table S2 in Supporting Information) based on their predicted profiles in the two treatments. Given our interest in early events, we assayed the following patterns: early induction in MSMs (ANCB01014346.1:6322‐7713; Fig. S3A, see Supporting Information); an *SSCRP* with sustained induction in MSMs (ANCB01009946.1:717‐1539; Fig. S3B); an unknown transcript showing early induction in GD12‐only microcosms (ANCB01002344.1:7484‐8547; Fig. S3C); and a *SSCRP* with early induction in MSMs (ANCB01002310.1:194‐864; Fig. S3D). In summary, these assays confirmed the early response expression profiles observed in the RNA sequencing data.

### *Trichoderma hamatum* GD12–*S. sclerotiorum* interactions elicit a biphasic transcriptional response

**Figure 6:**
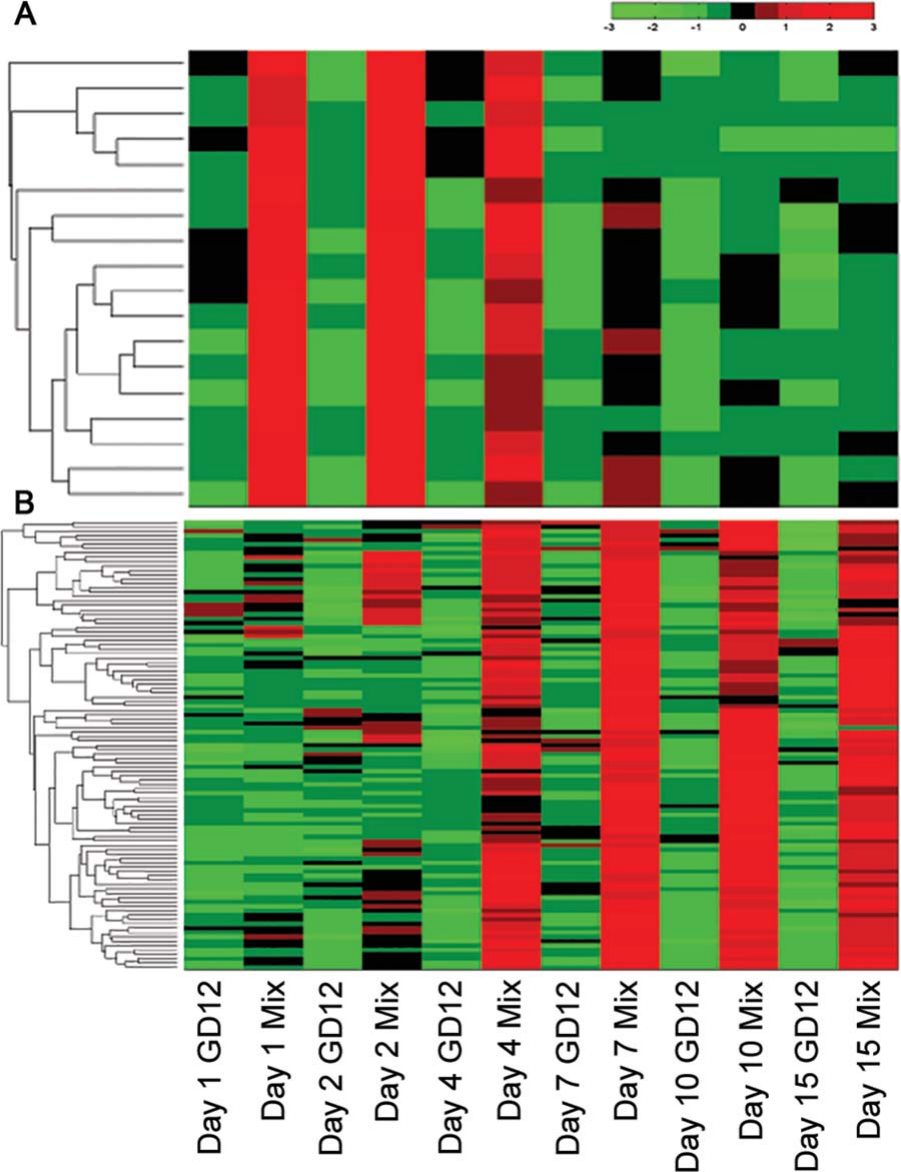
Hierarchal clustering reveals temporal deployment of specific sets of gene in mixed species microcosms. Clustering of genes based on normalized counts of reads using clustergram in MATLAB. (A) 18 genes with higher expression in the mixed species microcosms on 1 d, 2 d and 4 d. (B) 103 genes with higher expression in the MSMs on 4 d, 7 d, 10 d and 15 d.

We next focused on features that distinguished the antagonistic interaction using hierarchical clustering, and identified two distinct sets of genes up‐regulated by GD12 in response to *S. sclerotiorum* at early (1–4 days) and late (4–15 days) time points (Fig. 6). Eighteen genes showed higher expression during antagonism at early time points, two of which are hypothesized to have roles in the breakdown of the fungal cell wall and membrane. These two genes are orthologues of *T. hamatum* strain LU593, which were previously shown to be up‐regulated during confrontation with *S. sclerotiorum*: the alkaline proteinase *prb1* (Steyaert *et al*., 2004) and a metalloendopeptidase (Carpenter *et al*., 2005). These studies thus further validate our data, demonstrating commonality in *Trichoderma* transcriptional responses to biocontrol function, and support our prediction that this cluster defines a new set of antagonism‐specific genes. Notably, within the cluster, seven of the 18 genes encode strong homology to major facilitator superfamily (MFS) transporters and oligopeptide transporters. Given the prominent role of transporters in the active mobilization of secondary metabolites involved in antagonism (and/or roles in protection of the antagonist), the unexpectedly small number (Fig. 5C), but pronounced expression levels, of transporter genes associated with biocontrol at early time points is indicative of important functional roles early in antagonistic interactions with *Sclerotinia*. Other genes in this co‐expressed cluster included an oxidoreductase, a methyltransferase, two short‐chain dehydrogenase genes, a glycoside hydrolase, an HSCARG dehydrogenase [involved in NADPH sensing (Zhao *et al*., 2008) and regulation of H2A ubiquitination (Hu *et al*., 2014)] or chaperone proteins, all implicated in the modification of metabolites or gene regulation. The fact that 16 of the 18 genes in the MSM early response cluster have predicted functions (enzymatic and transport) implies that modification of small molecules/biosynthetic enzymes is a key early event in the establishment of GD12 biocontrol capability.

In addition to this early transcriptional response associated with active antagonism of *S. sclerotiorum* (Fig. 1), a second phase of transcriptional reprogramming in MSMs relative to GD12‐only microcosms was evident from 4 to 15 days, which may contribute to the enhanced PGP observed experimentally (Fig. 1). Six of the 103 genes up‐regulated during the later time points in MSMs were unique to GD12 and a further 23 encoded hypothetical *Trichoderma*‐specific proteins, suggesting that antagonism recruits genes capable of the synthesis of novel bioactive compounds. For 74 of the 103 genes up‐regulated late in MSMs, a functional inference was possible, revealing the recruitment of quite different complements of genes between the early and late phases of GD12 antagonistic interactions. Notable were genes with prominent roles in metal acquisition and carbon utilization, indicative of saprotrophic colonization or rhizosphere interactions. These genes include a PiT family inorganic phosphate transporter, four copper transporters, five hydrolases, a gene predicted to enable unusual carbon/nitrogen source utilization and three iron siderophore transporters. The latter reinforces the importance of siderophores, not only to the saprotrophic lifestyle, but also to biocontrol and rhizosphere colonization. Siderophore production has previously been linked to the biocontrol and PGP capabilities of the bacterium *Bacillus subtilis* CAS15 (Yu *et al*., 2011); however, this study is the first to implicate the importance of siderophores in fungal‐mediated biocontrol.

At the later time points in the MSMs, a number of genes whose products are associated with protective functions were induced. These genes encoded five MFS transporters, one fluconazole resistance protein, two trichothecene 3‐*O*‐acetyltransferases, which have been shown to protect against mycotoxins (Kimura *et al*., 1998), and two nitric oxide and mycotoxin‐protective dioxygenases. In addition, genes with potential functional roles in the production of lipids (essential precursors in secondary metabolite biosynthesis) and the production of antifungals were strongly over‐represented, including a squalene epoxidase involved in sterol biosynthesis, whose expression in *T. harzianum* is triggered by exposure to *Penicillium digitatum* cell wall extracts (Cardoza *et al*., 2006). Furthermore, genes encoding a perilipin‐like protein involved in lipid accumulation, a polyprenol reductase, a phenazine biosynthesis protein, a SUR2‐type hydroxylase/desaturase with a role in fatty acid biosynthesis and a gene with 53% identity to a gliotoxin biosynthetic gene were also over‐represented. Thus, collectively, late transcriptional reprogramming in MSMs appears to be strongly geared towards the production of secondary metabolites with protective or antifungal potential.

### SSCRPs expressed during PGP and biocontrol

**Figure 7:**
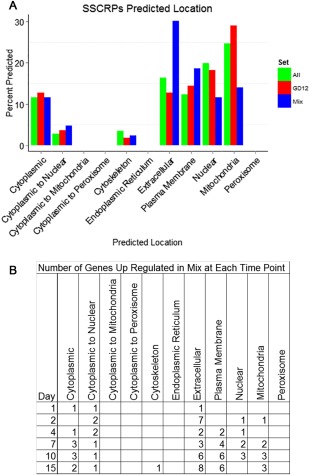
Predicted locations of small secreted cysteine‐rich proteins (SSCRPs). (A) Percentage location, as predicted by WoLF PSORT, of all predicted SSCRPs (green; Studholme *et al*. 2013). Those up‐regulated in mixed‐species microcosms are shown in blue and those up‐regulated in GD12‐only microcosms are shown in red. Predicted proteins lacking a methionine start could not be processed by WoLF PSORT, and are therefore not present. (B) Number of SSCRP genes up‐regulated at each predicted location at each time point in the mixed‐species microcosms.

SSCRPs are predicted effectors hypothesized to play important roles in *Trichoderma*‐mediated rhizosphere interactions (Moran‐Diez *et al*., 2015), the induction of systemic resistance (Lamdan *et al*., 2015) and antagonism (Atanasova *et al*., 2013). We identified 153 putative SSCRPs in the GD12 genome (Studholme *et al*., 2013), more than half (79) of which were expressed in GD12‐only and MSMs. Notably, of the 55 SSCRPs significantly up‐regulated in the GD12‐only microcosms (Table S3, see Supporting Information), the majority (∼60%) were expressed at 4 days or earlier. Sixteen were GD12 ‘unique’ (no annotation) and 14 represented ‘hypothetical’ proteins. Using WoLF PSORT to predict the cellular location, the majority of early GD12‐only induced SSCRPs were predicted as localized in the mitochondria (∼30%; Fig. 7A), with 13% extracellular and 15% plasma membrane localized. The role of these genes as effectors is currently under investigation.

In MSMs, 43 putative SSCRP‐encoding transcripts were significantly up‐regulated, 50% of which had no predicted annotation (Table S4, see Supporting Information). In contrast with the GD12‐only SSCRPs, which were primarily induced at 4 days or earlier, MSM SSCRPs were induced at later stages (4 days onwards, Fig. 7B), underlining the selective transcriptional dynamics under the two different conditions. Furthermore, WoLF PSORT predicted that ∼50% of MSM‐induced SSCRP genes encoded components likely to be extracellular or plasma membrane localized (Fig. 7A). Collectively, these data demonstrate that a number of GD12 genes encoding unique SSCRPs are differentially deployed under antagonistic interactions and are predicted to function in the immediate environment.

### antiSMASH analysis reveals potential for secondary metabolite production in *T. hamatum* GD12

Novel secondary metabolite production, most notably from non‐ribosomal peptide synthetases (NRPSs) and polyketide synthases (PKSs), is predicted to be important for both *Trichoderma* biocontrol and PGP (Baker *et al*., 2012; Mukherjee *et al*., 2012). Using antiSMASH (Blin *et al*., 2013), we identified 32 gene clusters in GD12, including NRPSs, type 1 PKSs and terpene synthases (Table S5, see Supporting Information). Strikingly, only five antiSMASH clusters were homologous to other species, further highlighting the capacity for novel metabolite production by GD12. Most surprising was the prediction that cluster 31 homologues were bacterial specific.

**Figure 8:**
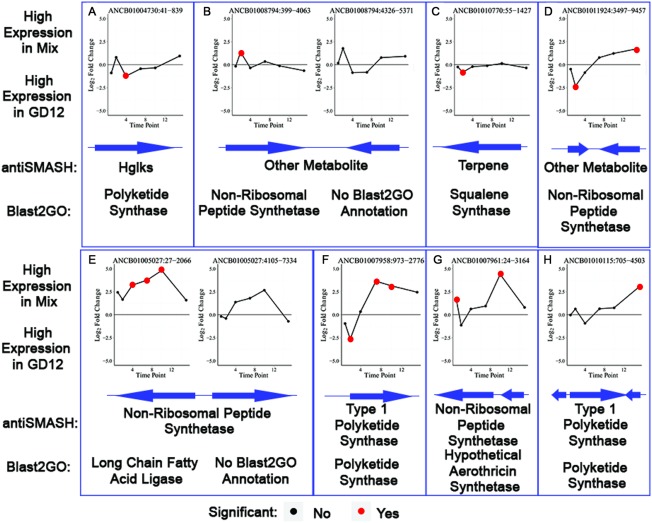
Expression trends for predicted genes with significant differences in expression between GD12‐only and mixed‐species microcosms on the following contigs: (A) ANCB01004730.1; (B) ANCB01008794.1; (C) ANCB010770.1; (D) ANCB01011924.1; (E) ANCB01005027.1; (F) ANCB01007958.1; (G) ANCB01007961.1; (H) ANCB010115.1. A positive fold change shows higher expression in the mixed‐species microcosms, whereas a negative fold change shows higher expression in the GD12‐only microcosms. Arrangement of the gene cluster on the contig as predicted by antiSMASH is shown below. Annotation of the cluster was by antiSMASH and gene prediction by Blast2GO.

We validated that antiSMASH predictions correlated with FGenesH predicted GD12 gene locations and mRNA‐seq reads, thus highlighting secondary metabolite biosynthetic gene clusters potentially linked to PGP and biocontrol. The cluster at ANCB01004730.1 contains a single gene, with BlastP hits to a PKS (β‐ketoacyl synthase), which shows increased expression at 4 days in GD12‐only microcosms (Fig. 8A). The contig ANCB01008794.1 contains a two‐gene cluster with homology to an NRPS from *T. atroviride* (the closest relative of *T. hamatum* for which complete genome sequence data are available), one of which showed significantly increased expression in the MSMs at 2 days (Fig. 8B). Transcripts from the predicted terpene synthase cluster at ANCB01010770.1 were significantly more abundant in GD12‐only microcosms at 2 days (Fig. 8C). antiSMASH predicted a two‐gene NRPS cluster in contig ANCB011924.1, whereas FGenesH predicted a single gene. In GD12‐only microcosms, this gene showed significantly higher expression at 2 days, whereas, in the MSMs, its expression was significant at 15 days (Fig. 8D), in line with our biphasic prediction. The long‐chain fatty acid ligase encoded in the NRPS two‐gene cluster predicted in contig ANCB01005027.1 showed a significant increase in transcription in MSMs at 4, 7 and 10 days (Fig. 8E). ANCB01007958.1, predicted to contain a type 1 PKS, showed increased expression in GD12‐only microcosms at 2 days and in MSMs at 7 and 10 days (Fig. 8F). Most notable, the NRPS‐containing contig ANCB01007961.1, predicted to encode aerothricin synthetase, showed significantly higher expression in MSMs at 1 and 10 days (Fig. 8G). Aerothricin synthetase is involved in the synthesis of the novel cyclic antifungal aerothricins, which inhibit the β‐1,3‐d‐glucan component of fungal cell walls (Pharmaceutica, 2002). antiSMASH predicted a three‐gene PKS cluster on ANCB01010115.1 (FGenesH predicted a single gene) with significantly higher reads in the MSMs at 15 days (Fig. 8H). This antiSMASH analysis reinforces the specific transcriptional differences between treatments in potential antimicrobial‐encoding secondary metabolism clusters, and in three instances demonstrates the late induction of both PKS (ANCB01007958.1, ANCB01010115.1) and NPRS (ANCB011924.1) clusters, indicating that novel secondary metabolism is part of the second biphasic response seen in MSMs during the elaboration of biocontrol and PGP (Fig. 6).

## Conclusions

This study was designed to capture the transcriptional reprogramming associated with the dual PGP and biocontrol uniquely exhibited by *T. hamatum* GD12 (Ryder *et al*., 2012; Studholme *et al*., 2013) in peat microcosms. GD12 is predicted to encode more than 40% unique proteins compared with its most closely related sequenced relative, *T. hamatum*. With ∼50% of the transcriptome being expressed in the six samples that covered a 15‐day period, we were able to capture the underlying transcriptional reconfiguration that is associated with functionally known and novel transcripts which are recruited to deploy the diverse armoury of cryptic chemistries that are potentially encoded in the GD12 genome. We identified biphasic response signatures and were able to assign specific transcriptional dynamics, and often transcripts of unknown function, to GD12 biocontrol and PGP strategies. The comparison of the GD12 ‘PGP transcriptome’ with that of its earlier inferred ‘biocontrol transcriptome’ has allowed us to identify new sets of antagonism‐specific genes and their temporal dynamics. Functional analysis demonstrates that key classes of genes are up‐regulated during lettuce growth promotion and during antagonistic interactions with the root‐infecting pathogen *S. sclerotiorum*, including transporter and oxidation–reduction genes, with a role for siderophores being prominent. We further identified the suite of SSCRP transcripts expressed during antagonism, and the transcripts associated with secondary metabolism clusters are predicted to be involved in the synthesis of a collection of antimicrobials. Collectively, our data indicate that the unique genomic potential of GD12 is deployed as an integral part of the establishment and maintenance of the biocontrol of a plant pathogen with a wide host range. This study provides the foundation to explore the functional contribution of this transcriptional reprogramming, particularly the novel GD12 components predicted to be involved in secondary metabolite formation, in inter‐species communication during the processes of PGP and plant disease control.

## Experimental Procedures

### Fungal strains

*Trichoderma hamatum* strain GD12 (GenBank accession no. AY247559; Ryder *et al*., 2012) and *S. sclerotiorum* strain M448 (courtesy of Jon West, Rothamsted Research, Harpenden, Hertfordshire, UK) were both cultured and maintained on potato dextrose agar at 26 ºC under a 16‐h light regime.

### Plant growth assays

Lettuce biocontrol assays were carried out essentially as described previously (Ryder *et al*., 2012; Studholme *et al*., 2013) with the following amendments. Sphagnum moss peat (Shamrock, Newbridge, Kildare) was supplemented with CaCO_3_ (Sigma, Sigma‐Aldrich Company Ltd., England, UK) at 0.5 g/L to adjust the pH to ∼5.8, and assays were carried out in six‐well plates (Nunc, Life Technologies Ltd, Paisley, UK) containing 50 g of peat per plate. Four peat microcosm treatments (illustrated in Fig. S1) were established as follows: (i) peat only (control); (ii) peat amended with *T. hamatum* GD12 inoculum alone (GD12 only); (iii) peat amended with *S. sclerotiorum* inoculum alone (*S. sclerotiorum* only); or (iv) peat amended with a mixture of both species (GD12 + *S. sclerotiorum*). Each treatment consisted of six six‐well plates, totalling 36 wells per treatment, two of which were pooled into one sample at each time point. Two lettuce (*Lactuca sativa* cultivar Webb's Wonderful) seeds were sown in each well and the microcosms were placed in a fully randomized design at 24 °C with a relative humidity of 90% and a 16‐h fluorescent light regime. The first emerging lettuce plant in each well was retained, whereas the other was removed. At six time points (1, 2, 4, 7, 10 and 15 days), six wells were selected at random from each treatment, above‐ground lettuce foliage was removed and the peat containing lettuce root and fungal biomass from two plugs was pooled. Each sample was immediately snap frozen in liquid nitrogen and stored at −80 °C prior to RNA extraction.

For dry weights, a replicate experiment was carried out as above. After 14 days, plants were harvested, washed and oven dried (75 ºC). Shoot and root weights of dried material were determined and the data were analysed using analysis of variance (ANOVA) and Tukey *post hoc* tests.

### Fungal RNA extraction from peat samples

Peat was homogenized to a granular powder using a blender (Waring, Hampshire, UK) in the presence of liquid nitrogen. From this material, a 2‐g aliquot of peat was used to extract total RNA using the PowerSoil® Total RNA Isolation kit (Mo Bio, Carlsbad, CA, USA) following the manufacturer's instructions. All 72 soil microcosm samples were quantified by both RNA gels and BioAnalyser traces (Agilent, Cheshire, UK) to assess quality. For samples falling below the threshold criteria, new RNA extractions were performed on the homogenized material. RNA from peat‐only microcosms was excluded from further analysis as the quantity of RNA extracted was negligible. Analysis proceeded on the remaining 54 samples.

### Library preparation and sequencing

Two microlitres of diluted ERCC RNA control spike‐in mix 1 or control spike‐in mix 2 (Life Technologies, Paisley, UK) were added to 1 µg of soil RNA, and the volume was adjusted to 50 µL with nuclease‐free water. Libraries were prepared using the TruSeq RNA application from Perkin‐Elmer (Utah, USA) for Illumina TruSeq RNA library preparation on a Sciclone next generation sequencing platform. Library quality and quantity were determined by LabChipGX high‐sensitivity assay (Perkin‐Elmer); 100‐bp paired‐end sequencing was then carried out using an Illumina HiSeq2500 (California, USA) platform with v3 reagents.

### qPCR validation of DEGs

Total RNA (1 μg) was DNAse treated with RNAse‐free DNAse (Ambion, Life Technologies, Paisley, UK) and, subsequently, first‐strand cDNA synthesis was carried out with Superscript III First Strand SuperMix (Invitrogen, Life Technologies, Paisley, UK) according to the manufacturer's instructions. Real‐time PCR was performed with the SensiFAST™ SYBR kit (Bioline, London, UK) in a Rotor‐Gene 6000 (Qiagen, Skelton House, Lloyd Street North, Manchester M15 6SH). In all cases, three technical replicates were run for each cDNA sample. Table S2 reports primer combinations and Fig. S3 reports validation results.

### Data analysis

The following Illumina pipeline processing was performed. Raw reads of low quality (phred score < 30) and adapter sequences were removed using Fastq‐mcf (Aronesty, 2011, accessed April 2014). External RNA Controls Consortium spike‐in control reads were removed using Bowtie (Langmead and Salzberg, 2012) and the quantification of expression was checked to demonstrate the quality of the library preparation. Filtered reads were aligned to the draft reference genomes for *T. hamatum* GD12 (Studholme *et al*., 2013), and unmapped reads were then aligned to the *S. sclerotiorum* strain 1980 (Amselem *et al*., 2011) using Tophat2 with standard settings (Trapnell *et al*., 2009).

Statistical analysis of differential expression was carried out using three separate software packages. HTSeq (Anders *et al*., 2014) was used for the quantification of reads at each predicted gene location. These counts were then analysed with DESeq2 (Love *et al*., 2014) and edgeR (Robinson *et al*., 2010) programs using a significance threshold of an adjusted *P* value of <0.05, representing a 5% false discovery rate, corrected for multiple testing using the Benjamini–Hochberg method (Benjamini and Hochberg, 1995). Cufflinks (Trapnell *et al*., 2010) was also used to quantify the transcripts, followed by differential expression analysis using CuffDiff2 (Trapnell *et al*., 2013), employing the same significance parameters as above.

The clustering of genes with similar normalized read counts, as determined by DESeq2, was carried out using the clustergram function in MATLAB. Functional predictions of genes were made using results from blastx (Altschul *et al*., 1990) alongside analysis with the Blast2GO software package (Conesa *et al*., 2005). Predictions of protein location were made using WoLF PSORT (Horton *et al*., 2007) for all translated gene predictions starting with a methionine. The identification of potential secondary metabolite biosynthetic pathways within the draft reference genome was achieved using antiSMASH version 2.0 (Blin *et al*., 2013) predictions.

### Availability of supporting data

Sequencing data supporting the results of this study can be found in the GEO repository under accession number GSE67909.

## Author Contributions

SS carried out all RNA sequencing data analyses, supported by DJS, and wrote the manuscript. MRG and KLC developed the experimental design. KLC and RW undertook the experimental assay. KLC extracted the RNA. KP and KM carried out the library preparation and sequencing. MdTZ carried out the qPCR validations. CRT and MRG advised throughout the investigation and, together with DJS and KLC, aided in manuscript preparation.

## Supporting information

Additional Supporting Information may be found in the online version of this article at the publisher's website:

Supporting Information

**Fig. S1** Overview of experimental microcosms.
**Fig. S2** Comparison of differential expression calling between DESeq2 and edgeR programs.
**Fig. S3** Reverse transcription‐polymerase chain reaction (RT‐PCR) validation of selected gene expression patterns between GD12‐only microcosms and mixed‐species microcosms
**Table S1** Summary of read statistics from each treatment replicate at each of the six time points
**Table S2** Primer combinations used for quantitative polymerase chain reaction (qPCR) validation of selected transcripts from GD12‐only and mixed‐species microcosms (MSMs). SSCRP, small secreted cysteine‐rich proteins.
**Table S3** Small secreted cysteine‐rich proteins (SSCRPs) up‐regulated in GD12‐only microcosms.
**Table S4** Small secreted cysteine‐rich proteins (SSCRPs) up‐regulated in mixed‐species microcosms.
**Table S5** Potential secondary metabolite‐producing gene clusters identified by antiSMASH.

**Supporting Material S1** Summary of significantly up‐regulated genes in GD12‐only microcosms at each time point.

**Supporting Material S2** Summary of significantly up‐regulated genes in mixed‐species microcosms at each time point.

**Supporting Material S3** Summary of significantly up‐regulated genes in GD12‐only microcosms at each time point, where expression in mixed‐species microcosms is zero.

**Supporting Material S4** Summary of significantly up‐regulated genes in mixed‐species microcosms at each time point, where expression in GD12‐only microcosms is zero.
