# Supplementary material for: Transcriptional reprogramming underpins enhanced plant growth promotion by the biocontrol fungus Trichoderma hamatum GD12 during antagonistic interactions with Sclerotinia sclerotiorum in soil
Source: Mol Plant Pathol. 2016 Jul 24;17(9):1425–41. doi: 10.1111/mpp.12429 (PMC6638342; doi:10.1111/mpp.12429)
Supplement: Supplementary file 1 — Supporting Information [file MPP-17-1425-s001.docx]

**Transcriptional reprogramming underpins enhanced plant growth promotion by the biocontrol fungus *Trichoderma hamatum* GD12 during antagonistic interactions with *Sclerotinia sclerotiorum* in soil**

Sophie Shaw, Kate Le Cocq, Konrad Paszkiewicz, Karen Moore, Rebecca Winsbury, Marta de Torres Zabala, David Studholme, Deborah Salmon, Christopher R. Thornton, and Murray R. Grant*

*** Correspondence:** Murray Grant, Biosciences, College of Life and Environmental Sciences, Stocker Road, University of Exeter, Exeter, EX4 4QG, UK

M.R.Grant@exeter.ac.uk

# Supplementary Data

Supplementary Materials 1 – Summary of significantly up-regulated genes in GD12 only microcosms at each time point

Supplementary Materials 2 – Summary of significantly up-regulated genes in mixed species microcosms at each time point

Supplementary Materials 3 – Summary of significantly up-regulated genes in GD12 only microcosms at each time point, where expression in mixed species microcosms is 0

Supplementary Materials 4 – Summary of significantly up-regulated genes in mixed species microcosms at each time point, where expression in GD12 only microcosms is 0

# Supplementary Figures and Tables – uploaded as single file
