# Supplementary material for: Transcriptional reprogramming underpins enhanced plant growth promotion by the biocontrol fungus Trichoderma hamatum GD12 during antagonistic interactions with Sclerotinia sclerotiorum in soil
Source: Mol Plant Pathol. 2016 Jul 24;17(9):1425–41. doi: 10.1111/mpp.12429 (PMC6638342; doi:10.1111/mpp.12429)
Supplement: Supplementary file 2 — Fig. S1 Overview of experimental microcosms. Fig. S2 Comparison of differential expression calling between DESeq2 and edgeR programs. Fig. S3 Reverse transcription‐polymerase chain reaction (RT‐PCR) validation of selected gene expression patterns between GD12‐only microcosms and mixed‐species microcosms Table S1 Summary of read statistics from each treatment replicate at each of the six time points Table S2 Primer combinations used for quantitative polymerase chain reaction (qPCR) validation of selected transcripts from GD12‐only and mixed‐species microcosms (MSMs). SSCRP, small secreted cysteine‐rich proteins. Table S3 Small secreted cysteine‐rich proteins (SSCRPs) up‐regulated in GD12‐only microcosms. Table S4 Small secreted cysteine‐rich proteins (SSCRPs) up‐regulated in mixed‐species microcosms. Table S5 Potential secondary metabolite‐producing gene clusters identified by antiSMASH. [file MPP-17-1425-s002.docx]

**Transcriptional reprogramming underpins enhanced plant growth promotion by the biocontrol fungus *Trichoderma hamatum* GD12 during antagonistic interactions with *Sclerotinia sclerotiorum* in soil**

**SUPPLEMENTARY FIGURES AND TABLES**
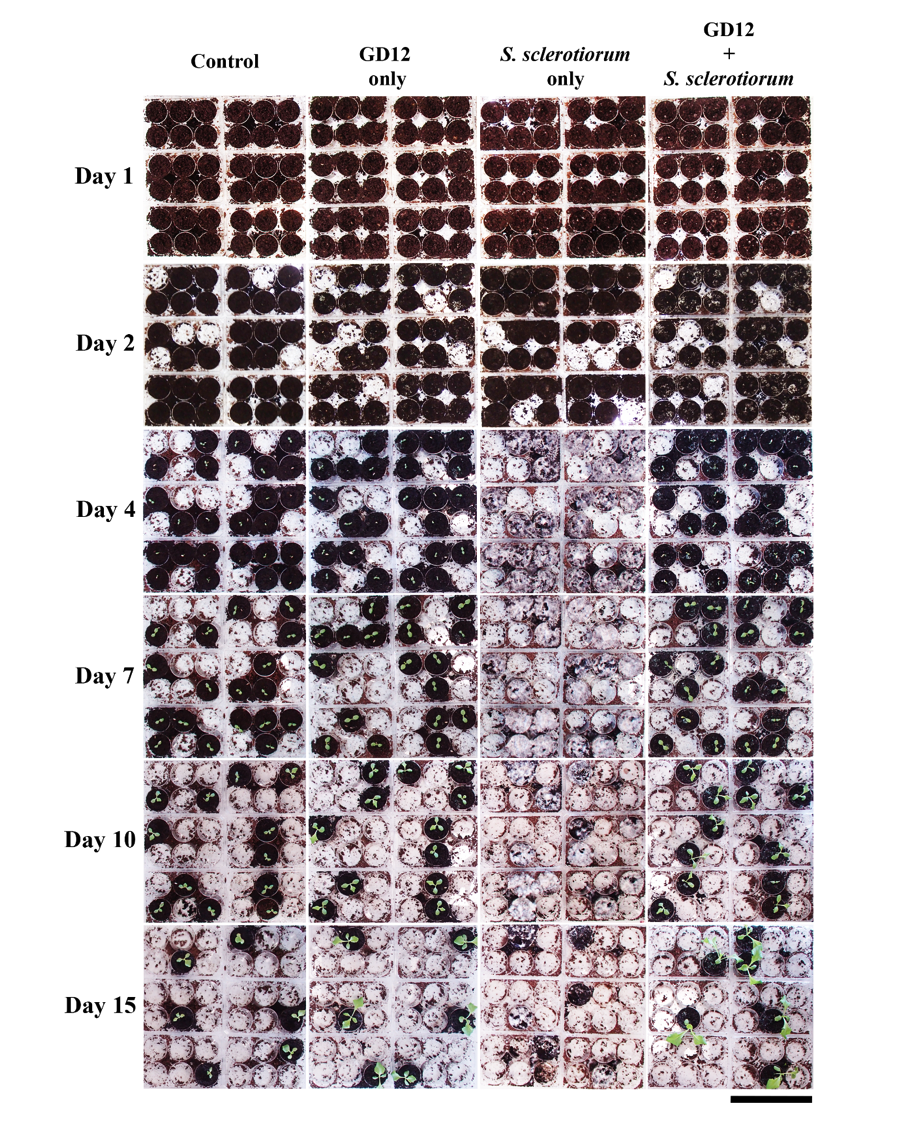
**Supplementary Figure 1.** Overview of experimental microcosms. Photographs of 6-well plates containing peat only (control), peat with *Trichoderma hamatum* GD12 only, peat with *Sclerotinia sclerotiorum* only, or peat with *Trichoderma hamatum* GD12 and *Sclerotinia sclerotiorum*. Replicate samples were removed at each time point and RNA extracted for sequencing. Red circle demonstrates single plant with atypical smaller growth. Scale bar = 12.7 cm.


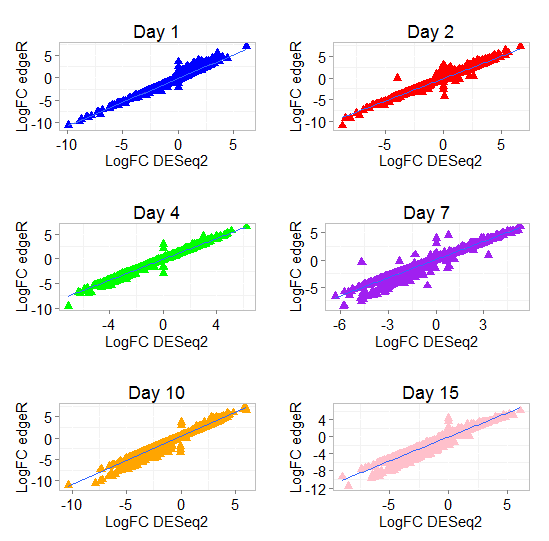


0.983

0.988

0.994

0.972

0.979

0.973

**Supplementary Figure 2.** Correlation of log-fold change in gene expression between *T. hamatum* GD12 only and mixed species microcosms as calculated by DESeq2 and edgeR at each time point.


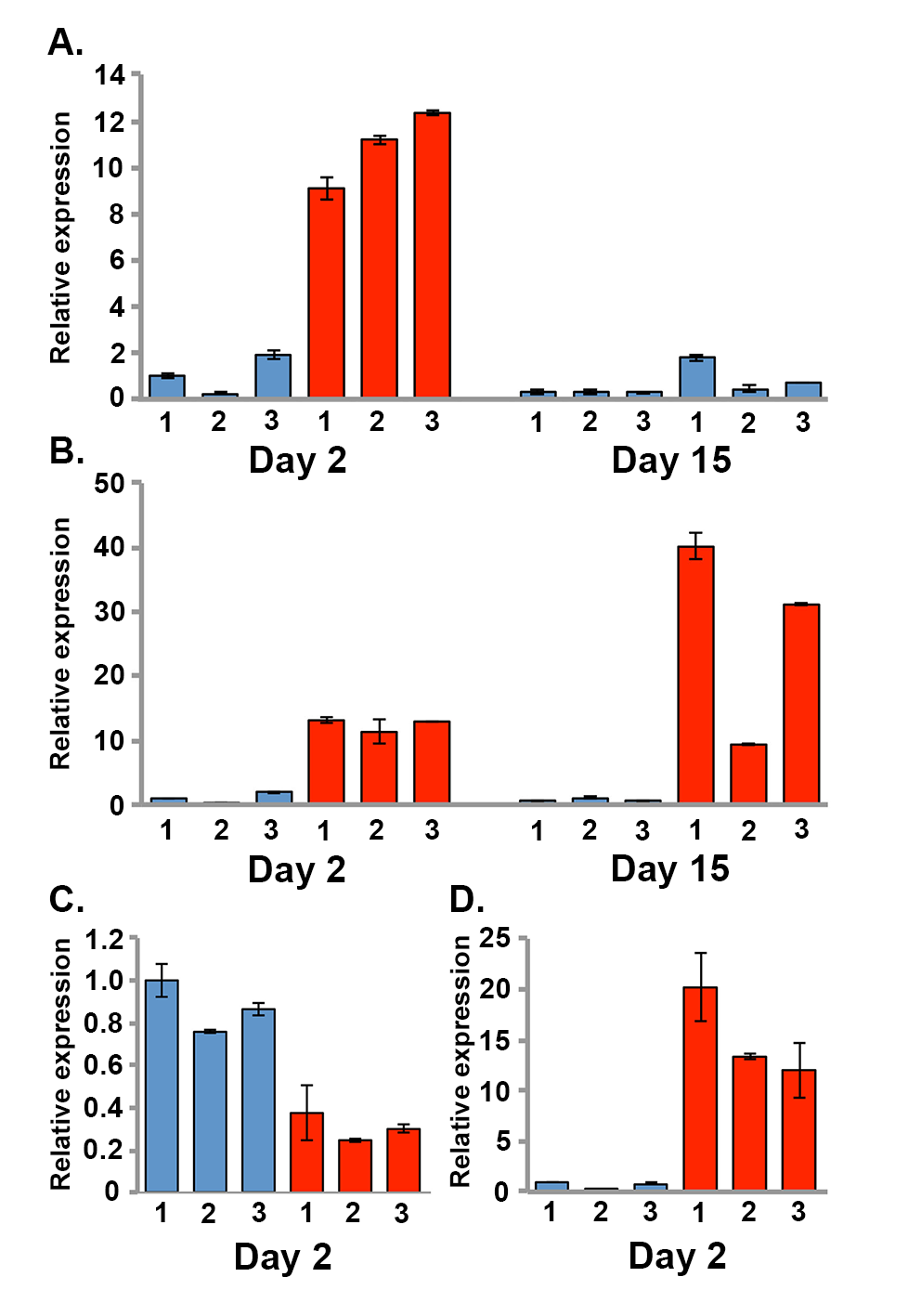


**Supplementary Figure 3.** qPCR validation of interesting representative mRNA-seq differential gene expression patterns between GD12 only microcosms and mixed species microcosms at d2 or d2 and d15. (A) Early induction of ANCB01014346.1:6322-7713 in mixed species microcosms. (B) Sustained induction of SSCRP ANCB01009946.1:717-1539 in mixed species microcosms, (C) Early induction of a gene ANCB01002344.1:7484-8547 in GD12 only microcosms, and (D) early induction of SSCRP ANCB01002310.1:194-864 in mixed species microcosms*.* RT-PCR was carried out using the same RNA samples as those used in the mRNA-seq time course. Three technical qPCR replicates were preformed on each biological replicate. To assess the efficiency of each primer pair combination, a 10-fold template dilution series was carried out in parallel. PCR efficiency was 1 ± 0.02. Quantitation was performed using the Two Standard Curves Quantification software provided (Corbett Research). Gene expression is recorded relative to the 40S Ribosomal Protein S3 gene (ANCB01000503.1:267-2035) which had relatively stable expression throughout the time course with log fold changes in expression of ‑0.029, 0.088, 0.007, 0.027. 0.110 and 0.056 at 1 d, 2 d, 4 d, 7 d, 10 d and 15 d respectively. Red bars are significantly different to levels for the first biological replicate of Day 2 GD12 only samples. Primer sequences are provided in Supplementary Table 2.

**Supplementary Table 1.** Summary of mRNA-seq read statistics from each treatments replicate at each of the 6 time points.

| **Sample** | **Total Reads** | **ERCC Reads** | **Reads Aligned to**  ***T. hamatum* GD12** | **Reads Aligned to**  ***S. sclerotiorum*** | **Reads Aligned to Both** |
| --- | --- | --- | --- | --- | --- |
| Day 1 *T. hamatum* GD12 only Rep 1 | 30367816 | 380968 | 27127800 | 38608 | 1691238 |
| Day 1 *T. hamatum* GD12 only Rep 2 | 12195499 | 178250 | 11217267 | 9540 | 427453 |
| Day 1 *T. hamatum* GD12 only Rep 3 | 23644477 | 453692 | 22012259 | 9413 | 453055 |
| Day 1 Mixed Rep 1 | 18694768 | 135838 | 14318383 | 2854826 | 1030625 |
| Day 1 Mixed Rep 2 | 13801283 | 327272 | 11625857 | 1383094 | 101133 |
| Day 1 Mixed Rep 3 | 14268740 | 97254 | 11747403 | 1906990 | 191210 |
| Day 1 *S. sclerotiorum* only Rep 1 | 13516767 | 272384 | 120132 | 12487235 | 113531 |
| Day 1 *S. sclerotiorum* only Rep 2 | 10740583 | 241369 | 517887 | 8471498 | 501884 |
| Day 1 *S. sclerotiorum* only Rep 3 | 25260340 | 525031 | 212341 | 22818731 | 201874 |
| Day 2 *T. hamatum* GD12 only Rep 1 | 28687803 | 276179 | 22205505 | 69483 | 2369994 |
| Day 2 *T. hamatum* GD12 only Rep 2 | 27218525 | 186212 | 22702394 | 39498 | 2261942 |
| Day 2 *T. hamatum* GD12 only Rep 3 | 12179366 | 144120 | 10039592 | 18360 | 917912 |
| Day 2 Mixed Rep 1 | 11362889 | 254261 | 9887287 | 243590 | 350682 |
| Day 2 Mixed Rep 2 | 13412373 | 316854 | 11630791 | 526783 | 729278 |
| Day 2 Mixed Rep 3 | 23936654 | 228517 | 20962453 | 434497 | 3416780 |
| Day 2 *S. sclerotiorum* only Rep 1 | 19850966 | 486942 | 777925 | 16028629 | 751706 |
| Day 2 *S. sclerotiorum* only Rep 2 | 18132262 | 150409 | 1699024 | 12147693 | 1652442 |
| Day 2 *S. sclerotiorum* only Rep 3 | 14266936 | 389724 | 686115 | 11207071 | 663307 |
| Day 4 *T. hamatum* GD12 only Rep 1 | 12507790 | 346286 | 9605250 | 18119 | 345931 |
| Day 4 *T. hamatum* GD12 only Rep 2 | 8054624 | 171057 | 5656778 | 10061 | 227685 |
| Day 4 *T. hamatum* GD12 only Rep 3 | 8713760 | 215002 | 6019660 | 17229 | 517022 |
| Day 4 Mixed Rep 1 | 12243444 | 205271 | 10467891 | 572840 | 167776 |
| Day 4 Mixed Rep 2 | 8797431 | 193110 | 7689293 | 245117 | 83100 |
| Day 4 Mixed Rep 3 | 8513449 | 401463 | 6990994 | 191709 | 219881 |
| Day 4 *S. sclerotiorum* only Rep 1 | 9203830 | 110294 | 353108 | 7361283 | 341297 |
| Day 4 *S. sclerotiorum* only Rep 2 | 15009715 | 116336 | 316110 | 13242150 | 303251 |
| Day 4 *S. sclerotiorum* only Rep 3 | 22696557 | 386691 | 509042 | 19311668 | 498086 |
| Day 7 *T. hamatum* GD12 only Rep 1 | 9497482 | 318613 | 5118162 | 18378 | 606295 |
| Day 7 *T. hamatum* GD12 only Rep 2 | 8601472 | 587854 | 4647884 | 16708 | 365432 |
| Day 7 *T. hamatum* GD12 only Rep 3 | NO SAMPLE |  |  |  |  |
| Day 7 Mixed Rep 1 | 10640175 | 292290 | 8995438 | 306916 | 186684 |
| Day 7 Mixed Rep 2 | 8626580 | 369091 | 6537756 | 565998 | 345062 |
| Day 7 Mixed Rep 3 | 11651330 | 274600 | 9495551 | 82163 | 498690 |
| Day 7 *S. sclerotiorum* only Rep 1 | 9861595 | 239669 | 469216 | 8320327 | 236044 |
| Day 7 *S. sclerotiorum* only Rep 2 | 9606314 | 235270 | 777057 | 4772175 | 739811 |
| Day 7 *S. sclerotiorum* only Rep 3 | 10198607 | 291224 | 702161 | 7745206 | 186544 |
| Day 10 *T. hamatum* GD12 only Rep 1 | NO SAMPLE |  |  |  |  |
| Day 10 *T. hamatum* GD12 only Rep 2 | 18554485 | 1078969 | 6384213 | 15255 | 482273 |
| Day 10 *T. hamatum* GD12 only Rep 3 | 6686161 | 623603 | 4106109 | 2350 | 77406 |
| Day 10 Mixed Rep 1 | 7615600 | 256627 | 6183884 | 21705 | 137244 |
| Day 10 Mixed Rep 2 | 13983047 | 456029 | 11420411 | 100801 | 279394 |
| Day 10 Mixed Rep 3 | 7089178 | 366857 | 1611158 | 2836352 | 302695 |
| Day 10 *S. sclerotiorum* only Rep 1 | 8497229 | 194284 | 1535706 | 5606087 | 101833 |
| Day 10 *S. sclerotiorum* only Rep 2 | 19657091 | 288241 | 1376790 | 11728459 | 1282487 |
| Day 10 *S. sclerotiorum* only Rep 3 | 6254960 | 204109 | 166476 | 4381167 | 125914 |
| Day 15 *T. hamatum* GD12 only Rep 1 | 14996590 | 164905 | 5575772 | 36378 | 1227743 |
| Day 15 *T. hamatum* GD12 only Rep 2 | 7039747 | 179117 | 2903360 | 16886 | 459475 |
| Day 15 *T. hamatum* GD12 only Rep 3 | 5790254 | 97936 | 2546689 | 13160 | 435297 |
| Day 15 Mixed Rep 1 | 29350474 | 533302 | 19919007 | 289671 | 1322148 |
| Day 15 Mixed Rep 2 | 7026963 | 279584 | 3945925 | 16149 | 328768 |
| Day 15 Mixed Rep 3 | 8334875 | 12332 | 5208560 | 12822 | 213758 |
| Day 15 *S. sclerotiorum* only Rep 1 | 10497112 | 343752 | 1447757 | 4795094 | 346965 |
| Day 15 *S. sclerotiorum* only Rep 2 | 3571383 | 275806 | 541301 | 1259419 | 95006 |
| Day 15 *S. sclerotiorum* only Rep 3 | 12098536 | 275851 | 4800264 | 4595432 | 567719 |

**Supplementary Table 2.** Primer combinations used for qRT-PCR validation (see Supplementary Figure 3).

| **Gene** | **Condition** | **Forward Primer^1^** | **Reverse Primer^1^** | **Product Size (bp)** | **Melting Temperature (FP/RP °C)** | **GC Content (FP/RP)** | **Complementary Binding^2^** |
| --- | --- | --- | --- | --- | --- | --- | --- |
| ANCB01014346.1:6322-7713 | Early induction in mixed species microcosms | TTGCCATCTTCACCCTTCCC | CGTTGCGATCTACAAGCAGC | 175 | 60/60 | 55/55 | None |
| ANCB01009946.1:717-1539 | Sustained induced SSCRP in mixed species microcosms | GTCAATCCAATCCACGCAGC | GCGGTGCATGGCAATGTTAT | 200 | 59.9/59.9 | 55/55 | None |
| ANCB01002310.1:194-864 | Early induced SSCRP in mixed species microcosms | ACCAGCTTTCCTTACTGGGC | GTTTGTGCTTCTGAAGGCGG | 179 | 60/60 | 55/55 | None |
| ANCB01002344.1:7484-8547 | Early induction in GD12 only microcosms | CAGAGGATTGCCGCAAGAGA | AGCCATGGACGCTCAAGAAA | 175 | 60.1/60 | 55/55 | None |
| ANCB01000503.1:267-2035 | Housekeeping Gene 40S Ribosomal Protein S3 | GATGGTGGTTGATGATGGCG | AATTCGACCCTGGCTCTTCG | 152 | 59.3/60.1 | 55/55 | None |

^1^ Primers designed using Primer3 (<http://primer3plus.com/cgi-bin/dev/primer3plus.cgi>)

2 Complementary binding tested using Life Technologies Multiple Primer Analyzer (<https://www.lifetechnologies.com/uk/en/home/brands/thermo-scientific/molecular-biology/molecular-biology-learning-center/molecular-biology-resource-library/thermo-scientific-web-tools/multiple-primer-analyzer.html>)

**Supplementary Table 3.** SSCRPs up-regulated in GD12 only microcosms.

| **Time Point** | **Gene Name** | **Wolf PSORT Prediction** | **Blast2GO Hit** | **Blastx** |
| --- | --- | --- | --- | --- |
| Day 1 | ANCB01008175.1 | Cytoplasmic | Related to bud7 protein (zinc ion binding) |  |
| Day 1 (and Day 2 Mix) | ANCB01011969.1 | Nuclear | Hypothetical protein (nucleic acid binding, nucleotide binding) | Hit 7 - Ribonucleoprotein Metarhizium anisopliae - 42% identity |
| Day 1 (and Day 10 & 15 Mix) | ANCB01012847.1 | Mitochondria | C6 transcription factor |  |
| Day 1, 4, 7, 10 & 15 | ANCB01001545.1 | (First a.a. not M) | No hit | Hit 4 - Exosome complex component mtr3 Beauveria bassiana - 78% identity |
| Day 2 | ANCB01001118.1 | Mitochondria | No hit | No hits |
|  | ANCB01001703.1 | Plasma Membrane | Hypothetical protein (metabolic process, "transferase activity, transferring acyl groups") | Hit 3 - 1-acyl-sn-glycerol-3-phosphate acyltransferase Verticillium dahliae - 71% identity |
|  | ANCB01002214.1 | Cytoplasmic to Nuclear | No hit | Hit 3 - Mediator of RNA polymerase II transcription subunit 16 Trichoderma reesei - 76% identity |
|  | ANCB01003286.1 | Mitochondria | Hypothetical protein (catalytic activity, metabolic process) | Hit 4 - Phosphoadenosine phosphosulfate reductase Colletotrichum gloeosporioides - 62% identity |
|  | ANCB01004508.1 | Plasma Membrane | Hypothetical protein (copper ion transport, copper ion binding, ATP binding, integral to membrane, cation-transporting ATPase activity) | Hit 4 - Putative Cu-ATPase Metarhizium anisopliae - 72% identity |
|  | ANCB01004610.1 | Nuclear | Hypothetical protein (asparagine-tRNA ligase activity, asparaginyl-tRNA aminoacylation, nucleic acid binding, ATP binding, cytoplasm) | Hit 4 - Asparaginyl-tRNA synthetase Trichoderma reesei - 94% identity |
|  | ANCB01006390.1 | Cytoplasmic | Hypothetical protein (hydrolase activity) | Hit 2 - Asparaginase family protein Metarhizium album - 58% identity |
|  | ANCB01007645.1 | Nuclear | No hit | Hit 2 - cis-Golgi transport protein particle complex subunit Trichoderma reesei - 91% identity |
|  | ANCB01007943.1 | Nuclear | Hypothetical protein (protein binding) | Hit 4 - WD domain, G-beta repeat containing protein Metarhizium acridum - 57% identity |
|  | ANCB01008121.1 | Mitochondria | Hypothetical protein (zinc ion binding) | Hit 4 - FHA domain containing protein Metarhizium acridum - 52% identity |
|  | ANCB01008359.1 | Mitochondria | hypothetical protein (integral to membrane) | Hit 4 - Clathrin-coated vesicle Colletotrichum orbiculare - 76% identity |
|  | ANCB01009018.1 | Mitochondria | No hit | No hits |
|  | ANCB01011938.1 | Nuclear | Hypothetical protein (sequence-specific DNA binding RNA polymerase II transcription factor activity, regulation of transcription from RNA polymerase II promoter, nucleus, zinc ion binding) | Hit 7 - C6 zinc finger domain-containing protein Colletotrichum higginsianum - 32% identity |
| Day 2 (and Day 4, 7, 10 & 15 Mix) | ANCB01000641.1 | Cytoplasmic | Tyrosine phosphatase protein |  |
| Day 2 (and Day 7 Mix) | ANCB01002468.1 | Nuclear | Hypothetical protein (actin binding) | Hit 10 - ADP-ribosylation factor Villosiclava virens - 42% identity |
|  | ANCB01007701.1 | Extracellular | Glycoside hydrolase family 54 protein |  |
|  | ANCB01008974.1 | Cytoplasmic | NADP-dependent mannitol dehydrogenase |  |
|  | ANCB01011604.1 | Cytoplasmic | No hit | Hit 2 - LmbE-like protein Trichoderma reesei - 71% identity |
| Day 2 (and Day 7, 10 & 15 Mix) | ANCB01000070.1 | Mitochondria | Telomere-linked helicase 1 |  |
|  | ANCB01000288.1 | Plasma Membrane | Ferric reductase transmembrane component 3 |  |
| Day 2 (and Day 10 Mix) | ANCB01013767.1 | Nuclear | No hit | No hits |
| Day 2 (and Day 10 & 15 Mix) | ANCB01003241.1 | Extracellular | No hit | No hits |
| Day 2 & 4 | ANCB01001545.1 | Plasma Membrane | Hypothetical protein (catalytic activity, metabolic process) | Hit 5 - Putative peroxisomal-coenzyme A synthetase-like protein Acremonium chrysogenum - 63% identity |
| Day 2 & 4 (and Day 10 Mix) | ANCB01009520.1 | Plasma Membrane | Hypothetical protein (single-organism transport; ion channel activity) | Hit 4 - Putative chloride channel protein 3 Torrubiella hemiterigena - 71% identity |
| Day 2, 4 & 15 | ANCB01003615.1 | Cytoplasmic | NADPH-P450 reductase |  |
| Day 2 & 15 | ANCB01003738.1 | Nuclear | Cytoplasmic tRNA 2-thiolation protein 2 |  |
|  | ANCB01005275.1 | (First a.a. not M) | No hit | All hypothetical proteins |
| Day 4 | ANCB01000931.1 | (First a.a. not M) | Hypothetical protein (regulation of transcription from RNA polymerase II promoter, zinc ion binding, sequence-specific DNA binding RNA polymerase II transcription factor activity, DNA binding, nucleus) | Hit 5 - C6 transcription factor Lichtheimia corymbifera - 37% identity |
|  | ANCB01002899.1 | Plasma Membrane | No hit | Hit 3 - 60S ribosomal protein L19 Trichoderma reesei - 76% identity |
| Day 4 (and Day 2 Mix) | ANCB01008151.1 | Extracellular | No hit | Hit 4 - Putative exo-beta- -glucanase protein Eutypa lata - 56% identity |
|  | ANCB01010792.1 | Extracellular | Hydrophobin |  |
| Day 4, 7, 10 & 15 | ANCB01002388.1 | Plasma Membrane | No hit | Hit 22 - Related to integral membrane protein Fusarium fujikuroi - 56% identity |
|  | ANCB01009821.1 | Mitochondria | Phosphoribosylformimino-5-aminoimidazole carboxamide ribotide isomerase |  |
| Day 4, 10 & 15 | ANCB01012043.1 | Extracellular | Glycoside hydrolase family 61 protein |  |
| Day 4 & 15 | ANCB01004039.1 | Nuclear | Cutinase transcription factor 1 |  |
| Day 7 & 15 | ANCB01012356.1 | Mitochondria | chch domain protein |  |
|  | ANCB01012810.1 | Mitochondria | 40s ribosomal protein s27 |  |
| Day 10 (and Day 2 Mix) | ANCB01010710.1 | Extracellular | No hit | All hypothetical proteins |
|  | ANCB01012991.1 | Mitochondria | No hit | Hit 3 - Putative S-adenosylmethionine-dependent methyltransferase Metarhizium anisopliae - 53% identity |
| Day 15 | ANCB01005474.1 | (First a.a. not M) | L-serine dehydratase |  |
|  | ANCB01000096.1 | Nuclear | No hit | No hits |
|  | ANCB01000786.1 | Mitochondria | C6 transcription |  |
|  | ANCB01001234.1 | Cytoplasmic | 3-hydroxyisobutyrate dehydrogenase |  |
|  | ANCB01001279.1 | Mitochondria | Fungal specific transcription factor domain-containing protein |  |
|  | ANCB01005153.1 | Plasma Membrane | Zn 2Cys6 transcription factor |  |
|  | ANCB01007437.1 | Mitochondria | Mitochondrial NADH-ubiquinone oxidoreductase 20 kd subunit |  |
|  | ANCB01009138.1 | Mitochondria | No hit | No hits |
|  | ANCB01011403.1 | Cytoskeleton | Dihydrouridine synthase |  |
|  | ANCB01014295.1 | Extracellular | Cellobiohydrolase i |  |
|  | ANCB01007323.1 | Mitochondria | NADH-ubiquinone oxidoreductase 23 kda subunit |  |
| Day 15 (and Day 1, 2 & 4 Mix) | ANCB01009685.1 | Cytoplasmic to Nuclear | Chorismate mutase |  |

**Supplementary Table 4.** SSCRPs up-regulated in mixed species microcosms.

| **GD12** | **Gene Name** | **Wolf PSORT Prediction** | **Blast2GO Hit** | **Blastx** |
| --- | --- | --- | --- | --- |
| Days 1, 2, 4, 10 & 15 | ANCB01009946.1 | Extracellular | No hit | All hypothetical proteins |
| Days 1, 2 & 4 (and Day 15 GD12) | ANCB01009685.1 | Cytoplasmic to Nuclear | Chorismate mutase |  |
| Days 1 & 10 | ANCB01007961.1 | Cytoplasmic | Hypothetical aerothricin synthetase 1 |  |
| Day 2 (and Day 1 GD12) | ANCB01011969.1 | Nuclear | Hypothetical protein (nucleic acid binding, nucleotide binding) | Hit 7 - Ribonucleoprotein *Metarhizium anisopliae* - 42% identity |
| Day 2 (and Day 4 GD12) | ANCB01008151.1 | Extracellular | No hit | Hit 4 - Putative exo-beta- -glucanase protein *Eutypa lata* - 56% identity |
|  | ANCB01010792.1 | Extracellular | Hydrophobin |  |
| Day 2 (and Day 10 GD12) | ANCB01010710.1 | Extracellular | No hit | All hypothetical proteins |
|  | ANCB01012991.1 | Mitochondria | No hit | Hit 3 - Putative S-adenosylmethionine-dependent methyltransferase *Metarhizium anisopliae* - 53% identity |
| Days 2, 4, 7, 10 & 15 | ANCB01000010.1 | Cytoplasmic to Nuclear | No hit | All hypothetical proteins |
| Days 2, 7, 10 & 15 | ANCB01002310.1 | Extracellular | Hydrophobin |  |
| Days 2 & 15 | ANCB01002501.1 | Extracellular | No hit | All hypothetical proteins |
| Days 4, 7 & 10 | ANCB01005399.1 | Nuclear | No hit | All hypothetical proteins - Trichoderma atroviride |
| Days 4, 7, 10 & 15 | ANCB01001013.1 | Extracellular | No hit | All hypothetical proteins |
|  | ANCB01006870.1 | Plasma Membrane | Prenyltransferase |  |
|  | ANCB01008382.1 | Plasma Membrane | No hit | All hypothetical proteins |
| Days 4, 7, 10 & 15 (and Day 2 GD12) | ANCB01000641.1 | Cytoplasmic | Tyrosine phosphatase protein |  |
| Day 7 | ANCB01009286.1 | - | Hypothetical protein (Mre11 complex; ATP binding; DNA repair) | Hit 3 - DNA repair protein RAD50 *Fusarium oxysporum* - 92% identity |
| Day 7 (and Day 2 GD12) | ANCB01002468.1 | Nuclear | Hypothetical protein (actin binding) | Hit 10 - ADP-ribosylation factor *Villosiclava virens* - 42% identity |
|  | ANCB01007701.1 | Extracellular | Glycoside hydrolase family 54 protein |  |
|  | ANCB01008974.1 | Cytoplasmic | NADP-dependent mannitol dehydrogenase |  |
|  | ANCB01011604.1 | Cytoplasmic | No hit | Hit 2 - LmbE-like protein *Trichoderma reesei* - 71% identity |
| Days 7 & 10 | ANCB01002630.1 | Mitochondria | Enoyl- hydratase isomerase |  |
| Days 7, 10 & 15 | ANCB01002082.1 | Extracellular | No hit | Hit 3 - VtaA26 *Haemophilus parasuis* - 61% identity |
|  | ANCB01008937.1 | Mitochondria | Alpha beta hydrolase family protein |  |
|  | ANCB01009496.1 | - | Serine threonine-protein kinase mps1 |  |
| Days 7, 10 & 15 (and Day 2 GD12) | ANCB01000070.1 | Mitochondria | Telomere-linked helicase 1 |  |
|  | ANCB01000288.1 | Plasma Membrane | Ferric reductase transmembrane component 3 |  |
| Day 10 | ANCB01005737.1 | Extracellular | No hit | Hit 6 - Signal peptide protein *Ophiostoma piceae* - 58% identity |
|  | ANCB01006736.1 | Plasma Membrane | Glycosyltransferase family 22 protein |  |
|  | ANCB01009223.1 | Nuclear | No hit | All hypothetical proteins |
|  | ANCB01012420.1 | - | Hypothetical protein (protein binding) | Hit 5 - NACHT and TPR domain protein *Ophiocordyceps sinensis* - 50% identity |
| Day 10 (and Day 2 GD12) | ANCB01013767.1 | Nuclear | No hit | No hits |
| Day 10 (and Days 2 & 4 GD12) | ANCB01009520.1 | Plasma Membrane | Hypothetical protein (single-organism transport; ion channel activity) | Hit 4 - Putative chloride channel protein 3 *Torrubiella hemiterigena* - 71% identity |
| Days 10 & 15 | ANCB01000300.1 | Cytoplasmic | dCMP deaminase |  |
|  | ANCB01000533.1 | Plasma Membrane | No hit | Hit 4 - Het-C-domain-containing protein *Trichoderma reesei* - 90% identity |
| Days 10 & 15 (and Day 1 GD12) | ANCB01012847.1 | Mitochondria | C6 transcription factor |  |
| Days 10 & 15 (and Day 2 GD12) | ANCB01003241.1 | Extracellular | No hit | No hits |
| Day 15 | ANCB01000786.1 | Plasma Membrane | No hit | All hypothetical proteins |
|  | ANCB01002348.1 | Cytoskeleton | Nonribosomal peptide synthase |  |
|  | ANCB01004984.1 | Plasma Membrane | Short chain dehydrogenase |  |
|  | ANCB01006979.1 | Extracellular | No hit | Fumarate hydratase *Trichoderma atroviride*- 91% identity |
|  | ANCB01007619.1 | Mitochondria | No hit | Hit 9 - Putative glutathione-dependent formaldehyde-activating protein *Eutypa lata* - 38% identity |
|  | ANCB01014118.1 | Extracellular | No hit | All hypothetical proteins |

**Supplementary Table 5.** Potential secondary metabolite producing gene clusters identified by antiSMASH.

| **Cluster** | **Contig** | **Type** | **Predicted Gene Location** | **Annotation** | **Species With Homologous Gene Clusters** |
| --- | --- | --- | --- | --- | --- |
| 1 | ANCB01001381.1 | Non ribosomal peptide synthetase | ANCB01001381.1:123-2421 | AMP-dependent Synthase and Ligase |  |
| 2 | ANCB01001383.1 | Non ribosomal peptide synthetase | ANCB01001383.1:111-5638 | Phosphopantetheine-binding domain-containing protein |  |
| 3 | ANCB01001384.1 | Non ribosomal peptide synthetase | ANCB01001384.1:90-2834 | Condensation domain-containing protein |  |
| 4 | ANCB01001472.1 | Non ribosomal peptide synthetase | ANCB01001472.1:48-5048 | AMP-dependent Synthase and Ligase |  |
| 5 | ANCB01001474.1 | Non ribosomal peptide synthetase | ANCB01001474.1:324-4373 | Condensation domain-containing protein |  |
| 6 | ANCB01001476.1 | Non ribosomal peptide synthetase | ANCB01001476.1:276-2636 | Biosynthetic Gene |  |
| 7 | ANCB01001479.1 | Non ribosomal peptide synthetase | ANCB01001479.1:119-5948 | AMP-dependent Synthase and Ligase |  |
| 8 | ANCB01001481.1 | Non ribosomal peptide synthetase | ANCB01001481.1:173-6273 | AMP-dependent Synthase and Ligase |  |
| 9 | ANCB01001483.1 | Non ribosomal peptide synthetase | ANCB01001483.1:269-7858 | Condensation domain-containing protein |  |
| 10 | ANCB01001798.1 | Other | ANCB01001798.1:90-850 | AMP-dependent Synthase and Ligase |  |
| 11 | ANCB01001799.1 | Other | ANCB01001799.1:3837-5159 | Phosphopantetheine-binding domain-containing protein |  |
| 12 | ANCB01001869.1 | Non ribosomal peptide synthetase | ANCB01001869.1:599-1593 | AMP-dependent Synthase and Ligase | *Trichoderma atroviride*  *Cordyceps miliataris* |
|  |  |  | ANCB01001869.1:3878-4130 | Other Gene |  |
|  |  |  | ANCB01001869.1:4538-6182 | Biosynthetic Gene |  |
|  |  |  | ANCB01001869.1:7838-8585 | Other Gene |  |
|  |  |  | ANCB01001869.1:8600-9275 | Other Gene |  |
| 13 | ANCB01001943.1 | Other | ANCB01001943.1:20-707 | Phosphopantetheine-binding domain-containing protein |  |
| 14 | ANCB01003031.1 | Other | ANCB01003031.1:5556-7210 | Phenylalanine-specific permease | *Trichoderma atroviride*  *Trichoderma virens*  *Trichoderma reesei*  *Metarhizium anisopliae*  *Metarhizium acridum*  *Fusarium oxysporum*  *Mycobacterium intracellulare*  *Mycobacterium intracellulare*  *Mycobacterium colombiense*  *Mycobacterium* sp. MOTT36Y |
|  |  |  | ANCB01003031.1:8600-12131 | AMP-dependent Synthase and Ligase |  |
| 15 | ANCB01004730.1 | Hglks | ANCB01004730.1:41-765 | Beta-ketoacyl synthase |  |
| 16 | ANCB01005027.1 | Non ribosomal peptide synthetase | ANCB01005027.1:68-2950 | AMP-dependent Synthase and Ligase | *Trichoderma atroviride*  *Trichoderma reesei*  *Trichoderma virens*  *Metarhizium anisopliae*  *Cordyceps miliataris*  *Metarhizium acridum*  *Verticillium dahliae*  *Aspergillus oryzae*  *Cyanothece* sp. PCC 8801  *Actinoplanes firuliensis* |
|  |  |  | ANCB01005027.1:3684-6941 | AMP-dependent Synthase and Ligase |  |
| 17 | ANCB01005261.1 | Type 1 Polyketide Synthase | ANCB01005261.1:1093-2231 | Short-chain dehydrogenase/reductase SDR | *Trichoderma atroviride*  *Trichoderma virens*  *Trichoderma reesei*  *Neurospora tetrasperma*  *Neurospora tetrasperma*  *Aspergillus kawachii*  *Glarea lozoyensis*  *Gloebacter violaceus*  *Stigmatella aurantiaca*  *Streptomyces lasaliensis* |
|  |  |  | ANCB01005261.1:2875-3583 | Other Gene |  |
|  |  |  | ANCB01005261.1:4087-5076 | Short-chain dehydrogenase/reductase SDR |  |
|  |  |  | ANCB01005261.1:6720-10195 | Beta-ketoacyl synthase |  |
| 18 | ANCB01006004.1 | Bacteriocin | ANCB01006004.1:1250-3433 | Biosynthetic Gene |  |
| 19 | ANCB01007767.1 | Other | ANCB01007767.1:191-2701 | NAD-dependent epimerase/dehydratase |  |
| 20 | ANCB01007958.1 | Type 1 Polyketide Synthase | ANCB01007958.1:598-2645 | Beta-ketoacyl synthase |  |
| 21 | ANCB01007961.1 | Non ribosomal peptide synthetase | ANCB01007961.1:181-2332 | Condensation domain-containing protein |  |
|  |  |  | ANCB01007961.1:3202-3270 | Other Gene |  |
| 22 | ANCB01008006.1 | Other | ANCB01008006.1:1141-3936 | AMP-dependent Synthase and Ligase |  |
|  |  |  | ANCB01008006.1:4159-4178 | Other Gene |  |
| 23 | ANCB01008794.1 | Other | ANCB01008794.1:443-2798 | AMP-dependent Synthase and Ligase |  |
|  |  |  | ANCB01008794.1:4154-5044 | Other Gene |  |
| 24 | ANCB01009261.1 | Terpene Synthase | ANCB01009261.1:276-2469 | Polyprenyl Synthetase |  |
|  |  |  | ANCB01009261.1:3706-3731 | Other Gene |  |
| 25 | ANCB01010115.1 | Type 1 Polyketide Synthase | ANCB01010115.1:858-1147 | Other Gene |  |
|  |  |  | ANCB01010115.1:1377-3807 | Beta-ketoacyl synthase |  |
|  |  |  | ANCB01010115.1:3921-4959 | Other Gene |  |
| 26 | ANCB01010770.1 | Terpene Synthase | ANCB01010770.1:55-1427 | Biosynthetic Gene |  |
| 27 | ANCB01011924.1 | Other | ANCB01011924.1:3262-3836 | Other Gene |  |
|  |  |  | ANCB01011924.1:6402-9431 | AMP-dependent Synthase and Ligase |  |
| 28 | ANCB01011933.1 | Non ribosomal peptide synthetase | ANCB01011933.1:2806-5297 | Phosphopantetheine-binding domain-containing protein |  |
|  |  |  | ANCB01011933.1:6041-6069 | Other Gene |  |
| 29 | ANCB01011948.1 | Non ribosomal peptide synthetase | ANCB01011948.1:534-2107 | Biosynthetic Gene |  |
| 30 | ANCB01013178.1 | Other | ANCB01013178.1:794-4604 | AMP-dependent Synthase and Ligase |  |
|  |  |  | ANCB01013178.1:5175-6128 | Short-chain dehydrogenase/reductase SDR |  |
|  |  |  | ANCB01013178.1:7691-10338 | Other Gene |  |
| 31 | ANCB01013319.1 | Non ribosomal peptide synthetase | ANCB01013319.1:537-2701 | AMP-dependent Synthase and Ligase | *Streptomyces albus*  *Nodularia spumigena*  *Paenibacillus larvae*  *Brevibacillus brecis*  *Bacillus pumilus*  *Acetonema longum*  *Bacillus pumilus*  *Brevibacillus laterosporus*  Uncultered soil bacterium  *Burkholderia glumae* |
|  |  |  | ANCB01013319.1:3338-3961 | Condensation domain-containing protein |  |
|  |  |  | ANCB01013319.1:6007-6094 | Other Gene |  |
| 32 | ANCB01014264.1 | Type 1 Polyketide Synthase | ANCB01014264.1:1915-8035 | AMP-dependent Synthase and Ligase |  |
